# Brain tumor is a sequence-specific RNA-binding protein that directs maternal mRNA clearance during the *Drosophila* maternal-to-zygotic transition

**DOI:** 10.1186/s13059-015-0659-4

**Published:** 2015-05-12

**Authors:** John D Laver, Xiao Li, Debashish Ray, Kate B Cook, Noah A Hahn, Syed Nabeel-Shah, Mariana Kekis, Hua Luo, Alexander J Marsolais, Karen YY Fung, Timothy R Hughes, J Timothy Westwood, Sachdev S Sidhu, Quaid Morris, Howard D Lipshitz, Craig A Smibert

**Affiliations:** Department of Molecular Genetics, University of Toronto, 1 King’s College Circle, Toronto, Ontario M5S 1A8 Canada; Donnelly Centre, University of Toronto, 160 College Street, Toronto, Ontario M5S 3E1 Canada; Department of Biochemistry, University of Toronto, 1 King’s College Circle, Toronto, Ontario M5S 1A8 Canada; Department of Biology, University of Toronto, Mississauga, 3359 Mississauga Road, Mississauga, Ontario L5L 1C6 Canada; Edward S. Rogers Sr. Department of Electrical and Computer Engineering, University of Toronto, 10 King’s College Road, Toronto, Ontario M5S 3G4 Canada; Department of Computer Science, University of Toronto, 40 St. George Street, Toronto, Ontario M5S 2E4 Canada

## Abstract

**Background:**

Brain tumor (BRAT) is a *Drosophila* member of the TRIM-NHL protein family. This family is conserved among metazoans and its members function as post-transcriptional regulators. BRAT was thought to be recruited to mRNAs indirectly through interaction with the RNA-binding protein Pumilio (PUM). However, it has recently been demonstrated that BRAT directly binds to RNA. The precise sequence recognized by BRAT, the extent of BRAT-mediated regulation, and the exact roles of PUM and BRAT in post-transcriptional regulation are unknown.

**Results:**

Genome-wide identification of transcripts associated with BRAT or with PUM in *Drosophila* embryos shows that they bind largely non-overlapping sets of mRNAs. BRAT binds mRNAs that encode proteins associated with a variety of functions, many of which are distinct from those implemented by PUM-associated transcripts. Computational analysis of *in vitro* and *in vivo* data identified a novel RNA motif recognized by BRAT that confers BRAT-mediated regulation in tissue culture cells. The regulatory status of BRAT-associated mRNAs suggests a prominent role for BRAT in post-transcriptional regulation, including a previously unidentified role in transcript degradation. Transcriptomic analysis of embryos lacking functional BRAT reveals an important role in mediating the decay of hundreds of maternal mRNAs during the maternal-to-zygotic transition.

**Conclusions:**

Our results represent the first genome-wide analysis of the mRNAs associated with a TRIM-NHL protein and the first identification of an RNA motif bound by this protein family. BRAT is a prominent post-transcriptional regulator in the early embryo through mechanisms that are largely independent of PUM.

**Electronic supplementary material:**

The online version of this article (doi:10.1186/s13059-015-0659-4) contains supplementary material, which is available to authorized users.

## Background

Post-transcriptional regulation of gene expression plays an essential role in numerous biological processes in a variety of cell types. One context in which post-transcriptional regulatory mechanisms are particularly important is the earliest stages of embryogenesis, during which the zygotic genome is transcriptionally quiescent, and development is directed by mRNAs and proteins that are produced from the mother’s genome and loaded into the oocyte during oogenesis [1]. In the early embryo, gene expression is controlled post-transcriptionally via regulation of mRNA translation, stability, and localization by *trans*-acting factors such as RNA-binding proteins (RBPs) and microRNAs (miRNAs), which bind to *cis*-acting sequences present in their target transcripts. The passing of developmental control from maternal to zygotic gene products, referred to as the maternal-to-zygotic transition (MZT), involves large-scale degradation of the maternally derived mRNAs, which is mediated by two different types of degradation machineries, one type dependent exclusively on maternally derived factors (referred to here as ‘maternal’ or ‘early’ decay machineries) and the other type dependent on zygotically expressed factors (referred to here as ‘zygotic’ or ‘late’ machineries) [1,2].

*Drosophila* Brain Tumor (BRAT) plays an essential role during the *Drosophila* MZT by repressing the translation of maternal *hunchback* (*hb*) mRNA in the posterior of the embryo [3]. BRAT is a member of the TRIM-NHL family of proteins, which are conserved among metazoans. Indeed, BRAT-mediated repression of *hb* has served as a model for understanding the mechanisms by which TRIM-NHL proteins function as post-transcriptional regulators. BRAT regulates *hb* expression in cooperation with Nanos (NOS) and the PUF-family RBP, Pumilio (PUM) [3]. In embryos from *pum*, *brat*, or *nos* mutant mothers, HB protein is ectopically expressed in the posterior of the embryo, leading to a loss of abdominal cell fates and embryonic lethality [3-8]. A role for TRIM-NHL proteins as post-transcriptional regulators and cell fate determinants is not limited to *Drosophila* (reviewed in [9,10]). For example, the mammalian TRIM-NHL protein, TRIM71, functions in the reprogramming of differentiated cells into induced pluripotent stem cells through its ability to bind to and inhibit translation of EGR1 mRNA [11] and a recent study has shown that TRIM71 can both repress translation and induce mRNA degradation [12].

In *Drosophila*, regulation of *hb* translation by the BRAT-PUM-NOS complex is mediated by two Nanos Response Elements (NREs) in the *hb* mRNA’s 3′UTR. Each NRE contains two sequence motifs, which are known as Box A and Box B. The Box B motif matches the PUM consensus-binding site, UGUANAUA where N = A/C/G/U, and is directly bound by PUM’s C-terminal PUF-homology domain, which is a conserved RNA-binding domain (RBD). An earlier model to explain *hb* regulation proposed that PUM and NOS make direct contact with the NREs and each other, while BRAT is recruited via its interaction with PUM and NOS proteins [3,5,13-16]. Recent work, however, has demonstrated that BRAT directly associates with sequences in and around *hb*’s Box A motifs in a PUM-independent manner via its C-terminal NHL domain [17]. That mammalian TRIM56 and TRIM71 are cross-linked to poly(A) RNA after exposure of cells to UV-irradiation [18,19] is consistent with the ability of BRAT to bind directly to RNA.

In addition to their role in repressing *hb* mRNA in early *Drosophila* embryos, PUM, BRAT, and NOS have been shown to cooperate in regulating other mRNAs in different cell types. For example, a PUM-NOS-BRAT complex controls motor neuron excitability through binding and regulation of *paralytic* (*para*) mRNA [20], and these three proteins cooperate in regulation of dendrite morphogenesis in the larval peripheral nervous system [21].

PUM and BRAT also function together, and likely independent of NOS, to repress *mad* and *dMyc* mRNAs and thereby promote germ-line stem cell differentiation during oogenesis [22]. In addition, PUM and NOS can regulate mRNAs independent of BRAT. For example, PUM and NOS act together, and without BRAT, to regulate *cyclin B* mRNA in primordial germ cells [3,23,24].

Experiments in tissue culture cells have demonstrated that PUM can mediate repression of reporter mRNAs independently of both BRAT and NOS [25]. Likewise, BRAT appears to exert functions independent of PUM. For example, in addition to defects in abdominal development in embryos from *brat* mutant mothers, mutations in *brat* cause over-proliferation of neuroblasts in the larval brain, which leads to the production of tumorous overgrowth [26-30] that can metastasize upon transplantation into the abdomens of adult flies [31]. Flies mutant for *pum*, however, do not share this neuroblast overgrowth phenotype, suggesting that it represents a function for BRAT independent of PUM. Moreover, BRAT can repress reporter transcripts in a PUM-independent manner in cell line-based assays [17], supporting the notion that BRAT can function independent of PUM.

Despite the aforementioned examples, however, the extent to which PUM and BRAT cooperate versus act independently remains unclear, and whether BRAT has any functions independent of PUM in regulation of endogenous targets *in vivo* is uncertain. Moreover, the spectrum of RNA sequences recognized by BRAT has not been defined, and the range of mechanisms that BRAT employs to regulate its targets has not been explored.

To address these questions, and to identify potential novel biological functions for PUM and BRAT in early embryos, we have carried out genome-wide identification of the mRNAs associated with PUM and with BRAT. Our results demonstrate that each RBP is associated with hundreds of mRNAs in early embryos, less than one-third of which are co-bound by both RBPs. In addition, through both computational analysis of BRAT-associated mRNAs as well as *in vitro* assays, we have identified a consensus RNA motif bound by BRAT, the functional significance of which was confirmed using luciferase reporter assays in *Drosophila* tissue culture cells. Gene ontology (GO) term analysis of the functions of PUM- and BRAT-associated mRNAs suggests a number of novel biological roles for PUM and BRAT in early embryos. Analysis of the translational status and stability of PUM and BRAT mRNA targets reveals that: (1) targets of both RBPs are translationally repressed; (2) PUM targets are degraded primarily during the late phase of the MZT; and (3) BRAT-associated mRNAs are degraded during both the early and late phases. Consistent with a role for BRAT in both translational repression and transcript degradation, a luciferase-reporter mRNA carrying BRAT-binding sites is subject to both forms of regulation. An *in vivo* role for BRAT in mRNA degradation was verified by an analysis of the transcriptome of embryos lacking functional BRAT protein, which revealed that BRAT mediates the degradation of hundreds of maternal mRNAs during the MZT, as part of both the ‘early’ and ‘late’ decay machineries. Taken together, our results provide the first insights into BRAT’s role as a global regulator of mRNA decay during the MZT via direct binding to a large number of maternal transcripts.

## Results and discussion

### Genome-wide identification of BRAT- and PUM-associated mRNAs in early *Drosophila* embryos

To identify BRAT- and PUM-associated mRNAs in early *Drosophila* embryos we performed RNA co-immunoprecipitations followed by microarray analysis (RIP-Chip). For the immunoprecipitations, we first generated synthetic antibodies against BRAT and PUM using phage display approaches (see Methods). We have previously demonstrated that such antibodies, expressed and purified from *E. coli* as antigen-binding fragments (Fabs), can be used in RIP experiments to identify RBP-associated mRNAs [32,33]. After confirming the ability of these antibodies to immunoprecipitate BRAT and PUM by western blot (Additional file 1), we carried out RIP-Chip experiments using extracts prepared from wild-type embryos collected 0 to 3 h post egg-laying. Associated mRNAs were defined as those that, across three biological replicates, had a false discovery rate (FDR) of less than 5% and an average enrichment of at least 1.5-fold in the BRAT or PUM RIP-Chips compared to negative control RIP-Chips using synthetic antibody C1 [32,33].

**Figure 1:**
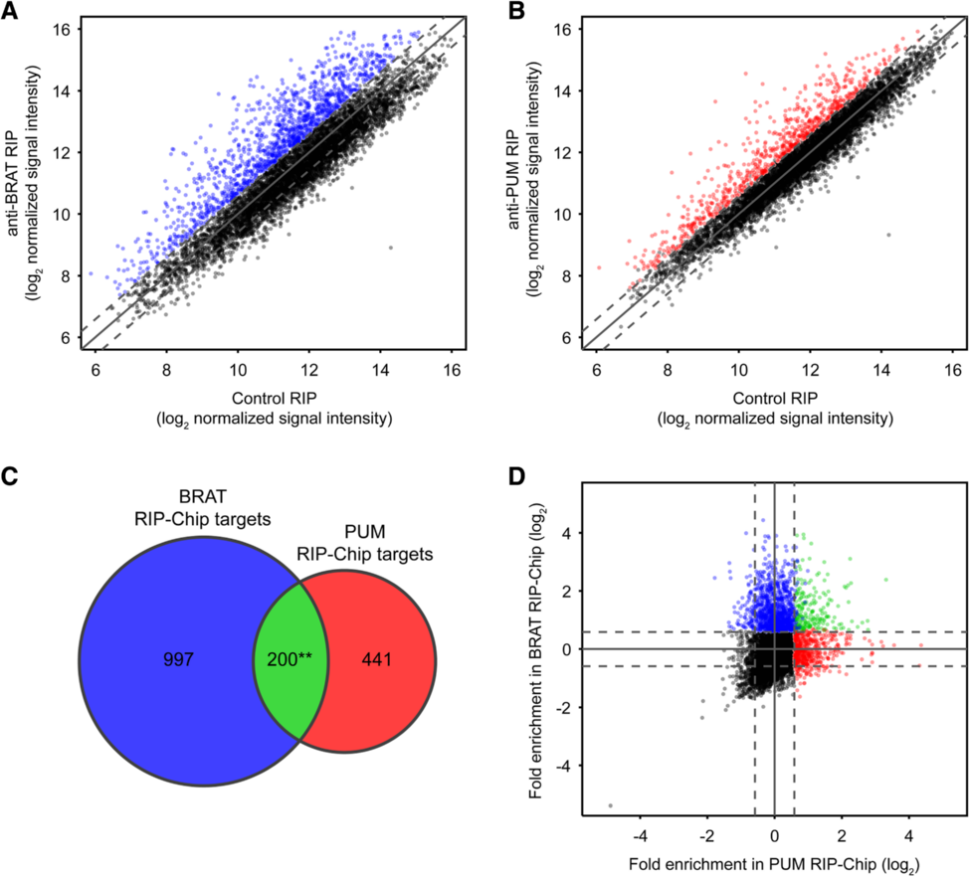
BRAT and PUM each associate with hundreds of mRNAs in early *Drosophila* embryos. **(A)** BRAT associates with mRNAs from 1,197 genes, and **(B)** PUM associates with mRNAs from 641 genes. Plots show RMA-normalized signal intensity of all transcripts represented on the microarray that were defined as expressed in early embryos, in BRAT RIP or PUM RIP versus Control RIP. Values represent averages from three independent biological replicates. mRNAs with an average enrichment of at least 1.5-fold in the BRAT or PUM RIPs and with an FDR <5% are highlighted in blue or red, respectively. The solid diagonal line represents no enrichment, and dashed diagonal lines represent 1.5-fold enrichment or depletion. **(C)** Venn diagram demonstrating that there is a modest but statistically significant overlap between BRAT-associated and PUM-associated mRNAs. **Fisher’s exact test P value <10^−13^. **(D)** Plot of all transcripts represented on the microarray that were defined as expressed in early embryos, showing fold-enrichment in the BRAT RIP-Chip versus the PUM RIP-Chip. Transcripts associated exclusively with BRAT or PUM (that is, enriched >1.5-fold with an FDR <5%) are highlighted in blue and red, respectively, and transcripts associated with both BRAT and PUM are highlighted in green. Note that the majority of BRAT-associated transcripts show no indication of enrichment in the PUM RIP-Chip, and *vice versa*; this is further indicated by the negative Spearman’s correlation between the fold-enrichments from the two experiments when transcripts which are enriched in the control in both experiments (that is, the bottom left quadrant of the plot) and therefore expected to be correlated given that the same control was used in both cases, are excluded: Spearman’s rho = −0.188, P <10^−37^.

mRNAs corresponding to 1,197 genes and 641 genes were found to be associated with BRAT and PUM, respectively (Figure 1A and B; Additional files 2 and 3). We refer to these associated mRNAs as BRAT ‘targets’ and PUM ‘targets’. *hb* mRNA, previously defined as both a PUM and BRAT target mRNA in early embryos, was on both target lists, while *bicoid* mRNA, which has been identified as a PUM target [34], was on the PUM but not on the BRAT list. *Cyclin B* mRNA, a third well-characterized target of PUM [3,23,24,35], just missed the cutoff for the PUM-target list (1.4-fold enriched in the PUM RIP compared to the negative control, with an FDR <10%) and was not on the BRAT list. In addition to regulating *hb* mRNA in early embryos, BRAT has been reported as a putative post-transcriptional repressor of other mRNAs in different tissues [22,30,36]. One of these, *dMyc*, is expressed in 0 to 3 h embryos and was also on our BRAT list. Finally, 300 (47%) of our PUM-bound mRNAs overlapped with a list of transcripts previously identified to be associated with transgenically expressed PUM-RBD in whole ovaries [37] (Fisher’s exact test *P* value <10^−109^) (Additional file 4). Together these data lend confidence to our lists of BRAT- and PUM-associated mRNAs.

### BRAT and PUM are associated with largely non-overlapping sets of mRNAs

To define the extent to which PUM and BRAT share targets we compared our lists of associated mRNAs. As described earlier, cooperation between BRAT and PUM has been demonstrated in the case of translational repression of *hb* mRNA as well as a number of other transcripts and, although BRAT-independent functions for PUM and PUM-independent functions for BRAT have been identified, the extent to which PUM and BRAT cooperate globally is unknown. Of the 1,197 BRAT-associated mRNAs and 641 PUM-associated mRNAs that we identified, 200 were found to be associated with both RBPs, a statistically significant overlap (Fisher’s exact test *P* value <10^−13^; Figure 1C). Comparison of our BRAT-associated mRNAs to those identified as targets of transgenically expressed PUM-RBD in whole ovaries [37] yielded similar results: a statistically significant overlap of 190 mRNAs (Fisher’s exact test *P* value <0.01; Additional file 4). Eighty-six mRNAs were in common between the BRAT-bound, PUM-bound, and PUM-RBD-bound lists. Together, these results support the hypothesis that a significant number of target RNAs are co-regulated by BRAT and PUM.

Despite the statistically significant overlap of PUM-targets and BRAT-targets, the majority of BRAT-targets were not bound by PUM and *vice versa.* To assess whether this might constitute evidence that BRAT and PUM also function independently of each other in early embryos, we examined the correlation between the fold-enrichments of mRNAs in the BRAT RIP-Chip versus the PUM RIP-Chip. Strikingly, the vast majority of BRAT-associated mRNAs showed no evidence of being associated with PUM, and the vast majority of PUM-associated mRNAs showed no evidence of being associated with BRAT (Figure 1D). Indeed, if we removed from consideration the transcripts that were enriched in the control RIP in both experiments (and therefore tend to correlate since the same negative control was used in both cases), there was a strong and statistically significant *negative* correlation between the fold-enrichments of the remaining mRNAs (Figure 1D; Spearman’s rho = −0.188, *P* value <10^−37^). This is the opposite of what would be expected if the two proteins primarily bind together, clearly demonstrating that BRAT and PUM are most often found associated with different target mRNAs.

### Identification of enriched motifs in PUM-associated and BRAT-associated mRNAs

To identify the RNA *cis*-element(s) recognized by PUM and BRAT *in vivo*, an algorithm we developed previously [38] was used to search for any short contiguous sequences that are predicted to be accessible to binding and that are enriched in PUM or BRAT target mRNAs compared to negative control sets of co-expressed mRNAs that are not bound by the respective RBPs (see Methods).

**Figure 2:**
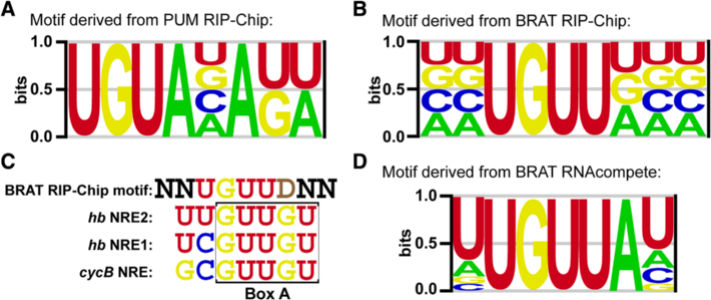
PUM- and BRAT-binding motifs. **(A)** Motif enriched among PUM-associated mRNAs, identified by *de novo* motif discovery. **(B)** Motif enriched among BRAT-associated mRNAs, identified by *de novo* motif discovery. **(C)** Comparison of the motif enriched among BRAT-associated mRNAs with the sequences of the ‘Box A’ motifs from the two *hb* NREs and the *cycB* NRE. Note that the Box A motif from *hb* NRE2 mediates repression by BRAT, but the Box A motifs from *hb* NRE1 and the *cycB* NRE do not (see text). **(D)** *In vitro* BRAT-binding motif determined by RNAcompete using purified GST-BRAT-NHL domain.

Analysis of our list of PUM-associated mRNAs yielded a motif, UGUANAKW (N = A/C/G/U; K = U/G; W = A/U) (Figure 2A) with a mean area under the receiver-operating characteristic (AUROC) of 0.78 on held-out data (see Methods for information on AUROCs and details of the cross-validation procedure, and Additional file 5 for the results). This motif is nearly identical to the previously reported PUM binding motif [5,15,16,37,39] and contains the invariant ‘UGUA’ sequence found among the binding sites of multiple PUF-family proteins from a variety of species [40]. Our *de novo* discovery of the known PUM binding motif strongly validates our list of PUM-associated mRNAs.

Motif discovery applied to our list of BRAT-associated mRNAs predicted a motif with the consensus ‘NNUGUUDNN’ (D = A/G/U) (Figure 2B) with a mean AUROC of 0.75 on held-out data (see Methods for details of the cross-validation procedure and Additional file 5 for the results). BRAT has recently been shown to directly associate with *hb* RNA, and its binding site has been mapped to the sequences in and around the *hb* NRE Box A [17]. Notably, our BRAT motif is a perfect match for the Box A site of the second *hb* NRE if the residues upstream of the Box A site are included (UUGUUGU; Figure 2C). In contrast, the Box A site of the first *hb* NRE lacks the first U found within the core UGUU of our motif (Figure 2C). These differences correlate with the reported differential behavior of the two NREs *in vitro* and *in vivo*: *in vitro*, the BRAT NHL domain binds more efficiently to the second *hb* NRE than the first, while in S2 cell reporter assays the Box A site of the second NRE is required for BRAT-mediated translational repression, whereas the Box A site of the first NRE has a negligible contribution to this repression [17]. Similarly, the NRE-like sequence in *cyclin B*, which lacks the core UGUU sequence of our BRAT motif, interacts with and functions through PUM and NOS, but not BRAT [3]. Taken together, these data strongly support the conclusion that the consensus motif identified by our computational analysis is likely to be the *bona fide* BRAT recognition site *in vivo*.

**Figure 3:**
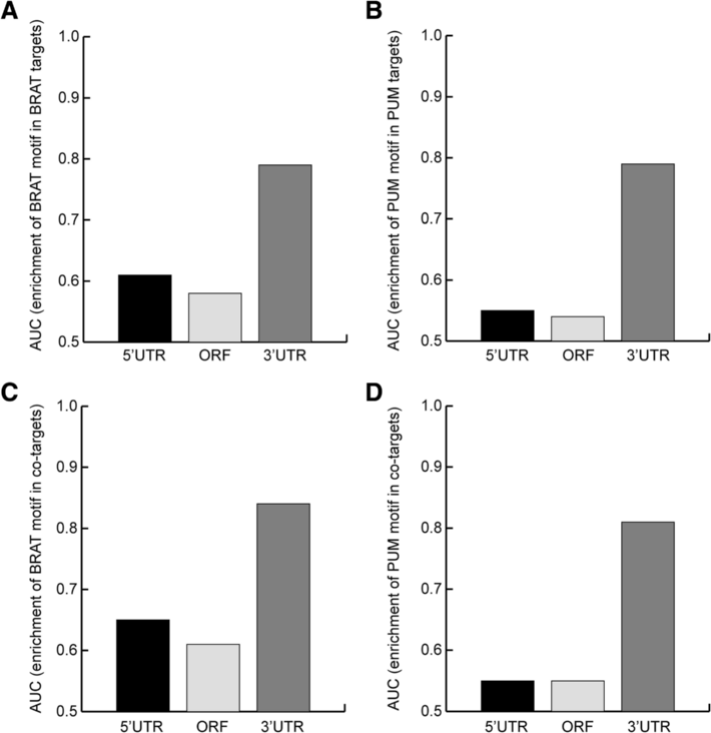
PUM and BRAT motifs are primarily enriched in the 3’UTRs of their associated mRNAs. AUROCs were calculated to determine the enrichment score of the PUM and BRAT motifs computationally determined from the RIP-Chip data in 5’UTRs, ORFs, and 3’UTRs of target transcripts. Assessment of the enrichment of **(A)** the BRAT motif among all BRAT targets, **(B)** the PUM motif among all PUM targets, **(C)** the BRAT motif among the subset of BRAT-PUM co-targets, and **(D)** the PUM motif among the subset of BRAT-PUM co-targets.

We next asked whether the consensus motifs we identified for PUM and BRAT tended to be present in the 5′ untranslated region (UTR), open reading frame (ORF), or 3′UTR of their target transcripts. To address this, we determined the AUROC for each consensus motif within each of these regions of the target transcripts compared to co-expressed non-targets. For both PUM and BRAT, their consensus motif was highly enriched in the 3′UTRs of their targets (Figure 3A and B; AUROC = 0.79). There was, in contrast, lower enrichment of the consensus motifs in either the 5′UTRs or ORFs (Figure 3A and B; AUROC = 0.55-0.65). A similar result was observed when we analyzed only those transcripts that are co-targets of both BRAT and PUM (Figure 3C and D). This indicates that both PUM and BRAT bind primarily to the 3′UTRs of their target transcripts.

**Figure 4:**
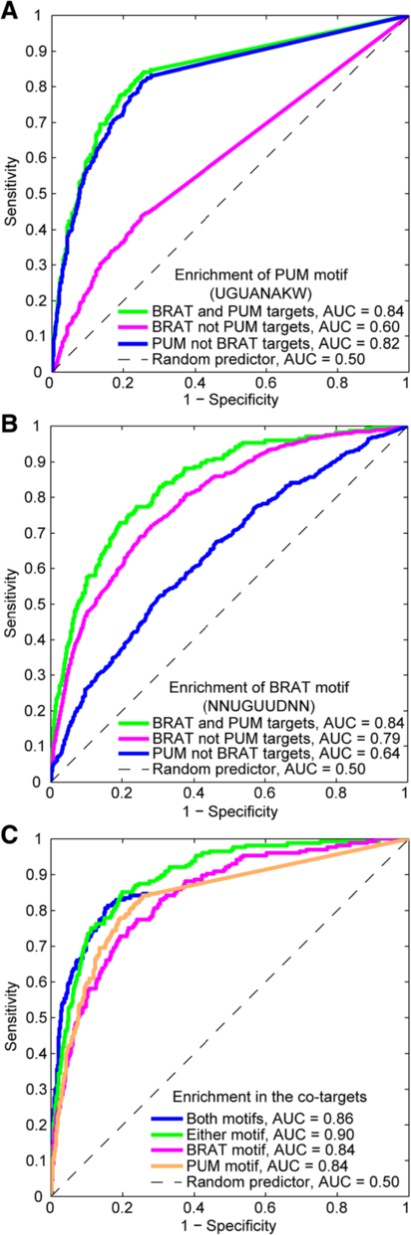
Enrichment of PUM and BRAT motifs among associated mRNAs. **(A)** AUROC plots showing how well the PUM motif determined from our RIP-Chip data distinguishes the following sets from co-expressed non-targets: BRAT and PUM co-targets, BRAT-only targets (BRAT not PUM targets), and PUM-only targets (PUM not BRAT targets). **(B)** AUROC plots showing how well the BRAT motif determined from our RIP-Chip data distinguishes the following sets from co-expressed non-targets: BRAT and PUM co-targets, BRAT-only targets (BRAT not PUM targets), and PUM-only targets (PUM not BRAT targets). **(C)** AUROC plots showing how well the presence of a BRAT motif alone (as determined from our RIP-Chip data), a PUM motif alone (as determined from our RIP-Chip data), either a BRAT or a PUM motif (Either motif), or both a BRAT and a PUM motif (Both motifs), distinguish the set of BRAT and PUM co-targets from co-expressed non-targets.

Finally, to assess our conclusion that BRAT and PUM primarily regulate different sets of transcripts, we assessed enrichment of the consensus motifs among targets bound by BRAT and PUM together, PUM only, or BRAT only. We found that the consensus PUM motif is highly enriched in the PUM and BRAT co-targets (AUROC = 0.84) as well as in the PUM-only targets (AUROC = 0.82), but is much less strongly enriched in the targets that were associated with BRAT only (AUROC = 0.60) (Figure 4A). Similarly, the consensus BRAT motif was highly enriched in the PUM and BRAT co-targets (AUROC = 0.84) as well as in the BRAT-only targets (AUROC = 0.79), but much less strongly in the PUM-only targets (AUROC = 0.64) (Figure 4B). These results support the conclusion that, while BRAT and PUM do co-associate with a subset of transcripts, they primarily bind different sets of mRNAs.

Interestingly, in this regard, the *pum* mRNA is high on the list of PUM targets (fold-enrichment = 20.5, FDR = 0%) and the *brat* mRNA is high on the list of BRAT targets (fold-enrichment = 6.2, FDR = 0%) but *pum* mRNA is not on the list of BRAT targets (fold-enrichment = 1.1, FDR = NA) and *brat* mRNA is not on the list of PUM targets (fold-enrichment = 1.04, FDR = 46%). We speculate that PUM and BRAT autoregulate their own, but not one another’s, mRNAs.

### *In vitro* identification of the spectrum of BRAT-binding sites

To complement the computational motif finding analysis of the BRAT targets identified by RIP-Chip, we assessed the spectrum of sites that are bound by BRAT *in vitro* using the RNAcompete assay [39,41]. This assay involves the incubation of a GST-tagged RBP or RBD of interest with an excess of a complex pool of approximately 240,000 30-41mer RNAs that contains at least 16 copies of each 9mer sequence and at least 310 copies of each 7mer. After capture of the protein on glutathione resin, co-purifying RNAs are identified using microarrays, allowing one to assess the binding specificity of the RBP.

Applying this approach to the BRAT NHL domain, which is BRAT’s RBD, we identified a motif that is very similar to the one we defined through computational motif finding applied to our RIP-Chip data: this RNAcompete motif has a core consensus UGUUA with a strong preference for a U residue both before and after the core (Figure 2D and Additional file 6). Taken together with our computational analysis of BRAT-associated mRNAs these data provide strong evidence that BRAT directly binds RNA, both *in vitro* and *in vivo*, through a motif carrying a core UGUU sequence.

### BRAT-binding sites direct BRAT-mediated repression in S2 tissue culture cells

**Figure 5:**
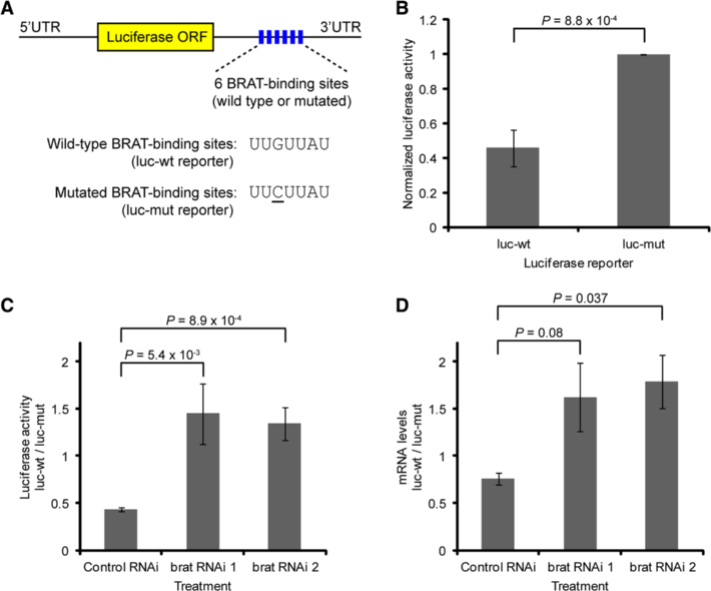
BRAT represses expression of reporter transcripts in S2 cells. **(A)** Schematic of luciferase reporters containing the Firefly luciferase ORF fused to a 3’UTR carrying either six wild-type (luc-wt) or six mutated (luc-mut) BRAT-binding sites. **(B)** Luciferase activity from each of the reporters was measured after transfection into S2 cells, and normalized to a Renilla luciferase control. Luciferase activity was reduced to approximately 40% by the wild-type sites compared to the mutated sites. **(C)** Normalized luciferase activity (Firefly/Renilla) from the luc-wt reporter relative to the luc-mut reporter was measured upon treatment with either control dsRNA or one of two dsRNAs directed against brat (Additional file 7 quantifies BRAT knockdown). RNAi-mediated knockdown of brat abrogated repression of the luc-wt reporter, resulting in an approximately three-fold increase in expression of the luc-wt reporter relative to the mutated reporter. **(D)** Normalized luciferase mRNA levels (Firefly/Renilla) from the luc-wt reporter relative to the luc-mut reporter were measured by RT-qPCR upon treatment with control dsRNA or either of the two dsRNAs directed against brat. The steady state level of the luc-wt reporter was approximately 75% that of the mutated reporter, indicating that BRAT-binding sites reduce steady-state RNA levels. RNAi-mediated knockdown of brat abrogated this repression, resulting in an approximately two-fold increase in expression of the wild-type reporter relative to the mutated reporter. Comparing the results in **(C)** and **(D)**, both the repression of the wild-type reporter relative to the mutated reporter and the increase in expression of the wild-type reporter upon BRAT knockdown, were less for steady state mRNA levels **(D)** than for luciferase activity **(C)**, indicating that BRAT regulates both mRNA stability and translational repression. Values represent average of three biological replicates **(B** and **C)** or two biological replicates **(D)** and error bars indicate standard deviation. Student’s t-test P values are indicated.

To assess the functional significance of the predicted BRAT-binding motif *in vivo* we used *Drosophila* S2 tissue culture cells in which we expressed luciferase reporter mRNAs carrying either an array of six wild-type or an array of six point-mutated BRAT-binding sites in their 3′UTRs (Figure 5A). The reporter with the wild-type array was repressed 2.2-fold relative to the reporter with the mutant array (Figure 5B, Student’s *t* test *P* <0.001). To verify that the observed repression was, indeed, mediated by BRAT, we carried out RNAi-mediated knockdown of BRAT (Additional file 7). Strikingly, repression was completely abrogated upon knockdown of BRAT (Figure 5C, Student’s *t* test *P* <0.01). We conclude that the BRAT-binding motif defined on the basis of our computational analysis of BRAT-bound transcripts as well as *in vitro* using RNAcompete, is indeed bound by BRAT *in vivo*, and mediates repression of the bound targets.

### Co-targets of PUM and BRAT contain binding sites for both RBPs

While our results indicate that PUM and BRAT largely function separately in mRNA regulation, there was a statistically significant overlap between their sets of target transcripts (Fisher’s exact test *P* value <10^−13^; Figure 1C). For this set of co-targets, the co-occurrence of the PUM and BRAT motifs had a similar predictive value (AUROC = 0.86) to the presence of either a PUM or a BRAT motif (AUROC = 0.90), and the presence of each motif alone (PUM motif AUROC = 0.84 and BRAT motif AUROC = 0.84) (Figure 4C). Thus, the PUM-BRAT co-targets have binding sites for both proteins, suggesting that they are directly bound by both RBPs and excluding models where either PUM or BRAT is indirectly recruited to these mRNAs via an interaction with the other protein. We note, however, that these results do not rule out the possibility that PUM and BRAT cooperate in the recognition of their co-targets. Indeed, *in vitro* experiments have demonstrated that the PUM RBD enhances the affinity of the BRAT RBD for the *hb* NREs, and *vice versa* [17].

### Functional analysis of all PUM-associated mRNAs

To gain insights into the biological and molecular processes regulated by PUM and BRAT, we asked whether our PUM- and BRAT-associated mRNAs are enriched for any particular functions. To assess this, we performed gene annotation enrichment analysis for our lists of PUM and BRAT target mRNAs using the DAVID functional annotation tool [42,43]. For this analysis, annotation terms enriched with a Benjamini *P* value of less than 0.1 or an FDR of less than 10% were considered significant, as in our previous analyses of the targets of Staufen (STAU) [33] and Smaug (SMG) [44].

Analysis of the entire set of PUM-associated mRNAs revealed enrichment for a number of developmental, cellular, and molecular functions (Table 1; Additional file 8). First, PUM-associated mRNAs were highly enriched for functions related to pattern formation, cell fate commitment, and morphogenesis during early embryogenesis. For instance, Gene Ontology (GO) terms such as ‘embryonic pattern specification’, ‘axis specification’, ‘blastoderm segmentation’, and ‘embryonic morphogenesis’ were highly enriched among PUM-associated mRNAs, consistent with previously reported analysis of transcripts associated with transgenically expressed PUM-RBD [37]. Our PUM-associated mRNAs that fell into these categories included ones with known roles in both anterior-posterior and dorsal-ventral axis formation in oocytes and early embryos (for example, *oskar* (*osk*), *nos*, *bicoid* (*bcd*), *caudal* (*cad*), *pum* itself, *gurken* (*grk*), *cactus* (*cact*), *easter* (*ea*), *swallow* (*sw*), *tolloid* (*tld*)), as well as mRNAs from gap genes, pair-rule genes, and segment polarity genes and their regulators (for example, *knirps* (*kni*), *Kruppel* (*Kr*), *hunchback* (*hb*), *caudal* (*cad*), *sloppy paired 1* (*slp1*), *even skipped* (*eve*), *cubitus interruptus* (*ci*), *frizzled 3* (*fz3*), *brother of tout-velu* (*botv*)). Moreover, PUM targets included mRNAs coding for Polycomb and trithorax group proteins (for example, *Polycomb* (*Pc*), *Posterior sex combs* (*Psc*), *female sterile (1) homeotic* (*fs(1)h*), *Enhancer of bithorax* (*E(bx)*)), which regulate the transcription of the homeotic genes, as well as regulators of homeotic gene activity (for example, *homothorax* (*hth*), *teashirt* (*tsh*)), and regulators of gastrulation (for example, *folded gastrulation* (*fog*)).

**Table 1:** **Summary of gene annotation enrichment analyses of BRAT- and PUM-associated mRNAs**

	**All PUM-associated mRNAs**	**mRNAs associated only with PUM**	**All BRAT-associated mRNAs**	**mRNAs associated only with BRAT**	**PUM and BRAT co-target mRNAs**
**Biological and developmental**					
Embryonic pattern specification	+++	++	.	.	.
Axis specification	++	.	+	.	+
Embryonic morphogenesis/gastrulation	++++	+++	.	.	.
Formation of organ boundary	++++	++++	.	.	.
Imaginal disc development/pattern formation	++++	+++	.	.	.
Oogenesis/ovarian follicle cell development	++	+	.	.	.
Neuroblast fate determination	+	.	.	.	+
Tube development/digestive system development/respiratory system development	++	+	.	.	.
Salivary gland development	+	+	.	.	.
Sensory organ development	+++	++	.	.	.
**Cellular**					
Cell motility/cell migration	+++++	+++	.	.	+
Neuron projection morphogenesis	+++	++	.	.	.
Cell division/asymmetric cell division	+	+	.	.	.
Cell adhesion	+	+	.	.	.
Regulation of growth/cell size	++	++	.	.	.
Regulation of apoptosis	+	.	.	.	.
**Molecular**					
Signal transduction	+++++	++++	+	.	+++
Regulation of transcription	++++	+++	.	.	+
Protein kinase activity	++	.	+	.	.
Phosphatase activity	.	.	+	.	.
Glycosyltransferase	++	+	.	.	.
Integral membrane proteins	++++	+	+++++	+++	+++
Glycoproteins	+++	++	.	.	.
Ion transport/homeostasis	.	.	+++	++	+
Phospholipid/phosphoinositide metabolism	.	.	+++	+++	.

+ FDR = 1% to 10%; ++ FDR = 0.1% to 1%; +++ FDR = 0.001% to 0.1%; ++++ FDR = 10^−5^% to 0.001%; +++++ FDR <10^−5^%; ‘.’ = not significant (FDR >10% and Benjamini *P* value >0.1). FDR values represent the most significantly enriched annotation term related to the function listed. Analyses were performed using the DAVID functional annotation tool [42,43]. For details see Additional file 8.

Taken together these results suggest that PUM’s role in pattern formation and morphogenesis during embryogenesis may extend beyond its well-characterized role in posterior patterning via translational repression of maternally derived *hb* mRNA. In support of this conclusion are reported defects in anterior development, including defects in head involution and mouth hook formation, which have been attributed to PUM’s regulation of *bicoid* mRNA at the anterior of the embryo [34]. Indeed, our analysis of the cuticles of embryos produced by females carrying the null-allele combination, *pum*^*ET7*^*/pum*^*Msc*^ [45-47], showed severely defective body pattern, including reduced or absent head skeleton (96%) and lack of both thoracic and abdominal segments (100%) (n = 23). With regard to these observed defects, it is interesting to note that ‘head segmentation’ is one of the pattern formation-related GO terms enriched among our list of PUM targets and includes the genes *homothorax* (*hth)*, *even skipped* (*eve*), *sloppy paired 1* (*slp1*), *teashirt* (*tsh*), *fushi tarazu* (*ftz*), *hb*, and *jing*.

In addition to these roles in embryonic pattern formation, PUM-associated mRNAs were enriched for GO terms related to pattern formation and morphogenesis during a variety of developmental processes that occur outside of the time window analyzed by our RIP-Chip. Such enriched GO terms included ‘oogenesis’, ‘formation of organ boundary’, ‘imaginal disc development’, ‘tube morphogenesis’, ‘gland development’, ‘sensory organ development’, and ‘neuroblast fate commitment’. As we have discussed previously for STAU [33] this, in part, reflects the fact that many of the genes and pathways that are involved in early embryonic processes are re-utilized later in development (for instance, 23 out of 50 genes annotated with the GO term ‘imaginal disc development’ are also annotated with GO terms related to embryonic pattern formation and morphogenesis). We therefore speculate that PUM may have important roles at a variety of developmental stages through the regulation of the same target mRNAs.

PUM-associated mRNAs were also enriched for a number of cellular-level functions. These included GO terms related to cell movement, such as ‘cell motion’ and ‘cell motility’. PUM has, indeed, been implicated in a variety of cell motility-related processes. For example, PUM has an essential role in primordial germ cell migration in embryos [23] and 35 of the 47 listed mRNAs coding for proteins with roles in cell motility are expressed in primordial germ cells. Six of these 35 have been classified as enriched in primordial germ cells [48], including *polar granule component* (*pgc*), *nos*, and *modifier of mdg4* (*mod(mdg4)*), all of which have also been specifically implicated in germ cell migration. PUM’s regulation of these transcripts may also contribute to its involvement in cell motility-related processes in other tissues. For instance, PUM has been shown to regulate dendritogenesis in the larval peripheral nervous system [49], and the GO terms ‘neuron projection morphogenesis’, ‘dendrite morphogenesis’, and ‘axonogenesis’ are all enriched among PUM targets.

PUM targets were also enriched for the GO terms ‘cell division’ and ‘asymmetric cell division’. PUM’s regulation of these mRNAs may contribute to the role of PUM in primordial germ cell mitosis, germ-line stem cell divisions [50], and the regulation of early nuclear divisions throughout the early embryo [35]. In addition, PUM targets were enriched for GO terms related to regulation of cell size and organism growth, as well as apoptosis. These targets included *Insulin-like receptor* (*InR*), *Akt1*, *Pten*, *Target of rapamycin* (*Tor*), and *p53*.

At the molecular level, PUM’s target mRNAs encoded proteins that were highly enriched for signal-transduction-related GO terms, such as ‘cell surface receptor linked signal transduction’ and ‘protein kinase activity’. Enrichment of these categories is consistent with analysis of transcripts associated with transgenically expressed PUM-RBD [37]. Our PUM-associated mRNAs included ones encoding components of a variety of signal transduction pathways: Jak-STAT, Notch, Wnt, insulin receptor, and MAPK signaling. With respect to MAPK signaling, PUM has recently been shown to regulate multiple components of the EGFR signaling pathway [51]. Our data may reflect a fairly general role for PUM in regulation of signal transduction.

PUM-associated mRNAs were also highly enriched for functions in ‘regulation of transcription’, including ones that encode transcriptional activators and repressors belonging to a number of families: HMG proteins, homeobox proteins, basic helix-loop-helix proteins, and Forkhead-family proteins. Also notable is the mRNA encoding Zelda/Vielfaltig (VFL), which is required for high-level activation of a subset of the zygotic genome [52-54]. Together, a role for PUM in regulation of signal transduction and transcription may reflect the mechanisms by which it regulates the developmental processes listed above.

Finally, PUM-associated mRNAs were enriched for GO terms such as ‘intrinsic to membrane’. This is, again, consistent with analysis of mRNAs associated with transgenically expressed PUM-RBD [37]. The products of our PUM-associated mRNAs localize to the plasma membrane and a variety of intracellular and organelle membranes. Particularly enriched were glycoproteins, glycosyltransferases, and proteins related to proteoglycan biosynthesis, suggesting a role for PUM in regulating glycoprotein synthesis and function.

### Functional analysis of all BRAT-associated mRNAs

We next performed gene annotation enrichment analysis on the entire set of BRAT-associated mRNAs (Table 1; Additional file 8). Several enriched GO terms overlapped with those enriched among PUM-bound mRNAs, including ‘axis specification’, ‘intracellular signaling cascade’, and ‘protein kinase activity’. Enrichment for genes with functions in axis specification is consistent with the fact that embryos produced by *brat*-mutant females have defects in abdominal segmentation [3], and suggests that misregulation of mRNAs in addition to *hb* may contribute to this phenotype.

BRAT targets were also enriched for mRNAs encoding membrane proteins, as indicated by enrichment of GO terms such as ‘intrinsic to membrane’, again similar to PUM. Indeed, this was the most highly enriched GO term among BRAT-associated mRNAs, and their encoded proteins localize to both the plasma membrane (68 genes were annotated with the GO term ‘plasma membrane’) and the membranes of a variety of organelles (73 genes were annotated with the GO term ‘organelle membrane’), including those of the endoplasmic reticulum, mitochondria, Golgi, nucleus, lysosome, and peroxisome. A particularly enriched subclass of these proteins, represented among BRAT-associated mRNAs but not PUM-associated mRNAs, is involved in ion transport, including ion channels, ATP-dependent ion transporters, and ion exchange factors.

In addition to membrane proteins themselves, BRAT target mRNAs were highly enriched for those encoding proteins that regulate the lipid component of membranes, as exemplified by enrichment for GO terms such as ‘phospholipid metabolic process’ and ‘phosphoinositide metabolic process’. These included a number of enzymes involved in fatty acid biosynthesis and phospholipid biosynthesis, as well as enzymes involved in the addition of glycophosphatidylinositol (GPI) anchors onto membrane-associated proteins. In addition, they included regulators of phosphoinositide signaling - both phosphatidylinositol kinases and phosphatidylinositol phosphatases.

### Functional analysis of mRNAs associated with both PUM and BRAT

Given that a number of related GO terms were enriched among both PUM and BRAT-associated mRNAs, we next asked whether any of these functions might be specifically regulated by PUM and BRAT acting together. To address this, we performed additional gene annotation enrichment analyses on the sets of mRNAs that were associated with: (1) PUM only; (2) BRAT only; and (3) both PUM and BRAT (Table 1; Additional file 8). This revealed that most of the enriched GO terms associated with all PUM targets were also enriched among the PUM-only targets. Similarly, the majority of the enriched GO terms associated with all BRAT targets were also enriched when one considered those mRNAs bound by BRAT only. These results suggest that most of the functions of PUM and BRAT can be regulated independently of one another. There are, however, notable exceptions, discussed below.

First, GO terms related to ‘neuroblast differentiation’ were significantly enriched among the set of BRAT-PUM co-targets. These GO terms were not enriched among the PUM-only or BRAT-only lists. Five BRAT-PUM co-targets were annotated with this term, including three of the four transcription factors that comprise the achaete-scute complex (*achaete* (*ac*), *scute* (*sc*), and *lethal of scute* (*l(1)sc*)), which have an important role in specifying neuroblast fate, as well as *hb* and *jumeau* (*jumu*). Although our RIPs were performed on embryonic stages prior to the formation of neuroblasts, this is particularly interesting given the well-characterized function of BRAT in regulating neuroblast differentiation. Taken together these data suggest a cooperative role for PUM and BRAT in neuroblast differentiation through regulation of the aforementioned transcripts.

Second, the GO terms ‘axis specification’, ‘zygotic determination of anterior/posterior axis, embryo’, and ‘signal transduction’ were enriched among BRAT-PUM co-targets. Several similar or identical terms were enriched among the PUM-only targets but not among the BRAT-only targets. This is consistent with the possibility that these functions are regulated by BRAT and PUM cooperatively.

### Analysis of the localization of PUM- and BRAT-associated mRNAs

We next analyzed the localization patterns of the different classes of target transcripts in order to define spatial aspects of PUM and BRAT function. To do so we used the localization pattern annotations in the Fly-FISH database [55,56]. Annotation terms and localization patterns enriched with an FDR of less than 10% were considered significant, as in our previous analyses of the targets of STAU [33] and SMG [44].

PUM-associated mRNAs were enriched for a variety of localization patterns (Table 2; Additional file 9). For example, PUM targets were highly enriched for transcripts localized to the ‘posterior’ and to the ‘pole plasm’ during embryonic stages 1 to 3. This is consistent with the requirement for PUM for normal primordial germ cell migration and mitosis, processes that PUM may control through post-transcriptional regulation of these posterior-localized mRNAs. PUM targets were also enriched for mRNAs with ‘perinuclear’ (at stages 1 to 3) and ‘cell division apparatus’ (at stages 4 to 5) localization patterns. These enrichments may reflect PUM’s role in cell division in a variety of contexts, as discussed above. BRAT-associated mRNAs, like PUM-associated mRNAs, were significantly enriched for genes with ‘posterior’ localization patterns, as well as ‘perinuclear’ localization (Table 2; Additional file 9).

**Table 2:** **Summary of Fly-FISH localization patterns enriched among BRAT- and PUM-associated mRNAs**

	**All PUM-associated mRNAs**	**mRNAs associated only with PUM**	**All BRAT-associated mRNAs**	**mRNAs associated only with BRAT**	**PUM and BRAT co-target mRNAs**
**Embryonic stages 1 to 3 localization patterns**					
Posterior localization	+	.	++	.	++
Nuclei-associated/Perinuclear	+	.	++	.	++
**Embryonic stages 4 to 5 localization patterns**					
Segmented/Gap-gene localization	+++	+++	.	.	.
Patterns indicative of zygotic transcription	+++++	++++	.	.	+
Perinuclear around yolk nuclei	+	+	.	.	.
Cell division apparatus	+	+	.	.	.
Apical enrichment	+	+	.	.	.
Pole cell enrichment	.	.	+	.	.
Yolk plasm localization	.	.	.	+	.

+ FDR = 1% to 10%; ++ FDR = 0.1% to 1%; +++ FDR = 0.001% to 0.1%; ++++ FDR = 10^−5^% to 0.001%; +++++ FDR <10^−5^%; ‘.’ = not significant (FDR >10%). FDR values indicated represent the most significantly enriched term related to the localization pattern listed. For details see Additional file 9.

Consistent with a role for PUM in regulation of pattern formation through control of zygotically transcribed mRNAs, PUM targets were also significantly enriched for both zygotically transcribed transcripts, generally, and ‘gap-gene’ localization patterns, in particular. In addition, PUM-associated mRNAs were enriched for ‘apical’ localization patterns.

We next assessed whether the observed enrichments also applied to PUM-only, BRAT-only, and/or PUM-BRAT co-target mRNAs (Table 2; Additional file 9). ‘Apical’, ‘cell division apparatus’, and ‘gap-gene’ localization patterns, as well as ‘zygotically transcribed’ transcripts in general, remained significantly enriched among PUM-only transcripts but not BRAT-only or PUM-BRAT co-targets. However, ‘perinuclear’ and ‘posterior localization’ were enriched among the PUM and BRAT co-targets but not among the PUM-only or BRAT-only targets. This enrichment for posterior localization suggests that BRAT may contribute to some of PUM’s functions in the development of pole cells.

Since both PUM and BRAT are ubiquitous whereas NOS is posterior localized, we speculate that NOS provides spatial specificity to the co-regulated transcripts, as is the case for *hb* mRNA. In support of this idea, a mutation in NOS has been shown to affect the recruitment of BRAT to the second *hb* NRE *in vitro* and translational repression of *hb in vivo*, without affecting either NOS or PUM association with the NRE [3], suggesting that NOS may stabilize BRAT’s interaction with the PUM-BRAT co-targets.

### BRAT- and PUM-associated mRNAs are translationally repressed and degraded during the maternal-to-zygotic transition

**Figure 6:**
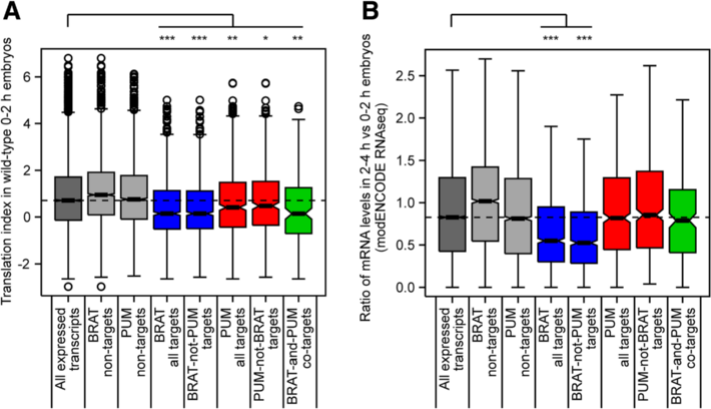
BRAT- and PUM-associated mRNAs are translationally repressed, and BRAT-associated mRNAs are degraded during the MZT. **(A)** Boxplots depicting the translation indices from wild-type 0 to 2 h embryos [44] for all expressed transcripts (that is, all transcripts found to be expressed in our 0 to 3 h embryos used for the RIP-Chip experiments), BRAT non-target transcripts, PUM non-target transcripts, all BRAT target transcripts, BRAT-not-PUM target transcripts (that is, all BRAT targets excluding BRAT-PUM co-targets), all PUM target transcripts, PUM-not-BRAT target transcripts (that is, all PUM targets excluding BRAT-PUM co-targets), and BRAT-and-PUM co-target transcripts. A higher translation index indicates more actively translated mRNAs. The dotted line represents the median translation index of all expressed transcripts detected in our 0 to 3 h RIP input embryo lysate. **(B)** Boxplots depicting the proportion of RNA present in 2 to 4 h embryos compared to 0 to 2 hour embryos, according to modENCODE RNAseq data available on FlyBase, for the same sets of transcripts described in **(A)**. Note that outliers (that is, transcripts for which the value is greater than 1.5 times the interquartile distance higher or lower than the upper or lower quartiles, respectively) are not shown to allow the y-axis to be magnified around the median values. The dotted line represents the median for all expressed transcripts detected in our 0 to 3 h RIP input embryo lysate. For both **(A)** and **(B)** Wilcoxon Rank Sum tests were performed to determine the significance of the differences between BRAT and PUM targets compared to ‘All expressed transcripts’: *P <0.01, **P <10^−5^, ***P <10^−27^.

To gain further insight into how PUM and BRAT function to regulate their target transcripts, we took advantage of previously published genome-wide datasets that defined the translational status or stability of mRNAs in early *Drosophila* embryos. We first assessed the efficiency with which PUM and BRAT target mRNAs are translated in early embryos. To do this, we made use of our published data that employed polysome gradients followed by microarray analysis to define, genome-wide, the efficiency with which mRNAs are translated in wild-type 0- to 2-hour-old embryos (Chen *et al.*, [44]). This was accomplished by calculating the ‘translation index’ for each mRNA by dividing the amount of that mRNA that is polysome-associated, and therefore likely to be translated, by the amount that is not polysome-associated, and thus not being translated [44]. We found that the translation indices of both PUM and BRAT target mRNAs were significantly lower than the translation indices of all of the transcripts assayed in our RIP experiments (Figure 6A; Table 3). This is consistent with the known roles of both PUM and BRAT as translational repressors. To further assess this conclusion, we also compared our target mRNAs to lists of mRNAs defined as ‘translationally active’ or ‘translationally inactive’ in 0- to 2-hour-old embryos by a second, independent, genome-wide polysome gradient-microarray study (Qin *et al.*, [57]). Again consistent with its role as a repressor, BRAT-associated mRNAs significantly overlapped with the list of translationally inactive transcripts and were significantly depleted of translationally active transcripts (Table 3; Additional file 10). PUM targets were not significantly correlated with translational repression based on this comparison (Table 3; Additional file 10).

**Table 3:** **Summary of comparisons of BRAT- and PUM-associated mRNAs to genome-wide mRNA translation and stability datasets**

	**All PUM-associated mRNAs**	**mRNAs associated only with PUM**	**All BRAT-associated mRNAs**	**mRNAs associated only with BRAT**	**PUM and BRAT co-target mRNAs**
**Translational repression**					
*Lower than expected translation index*					
Chen *et al.*, 2014 [44]^a^	+++	++	+++++	++++	+++
*Enriched for translationally inactive mRNAs*					
Qin *et al.*, 2007 [57]^b^	.	.	+++	+++	.
*Depleted of translationally active mRNAs*					
Qin *et al.*, 2007 [57]^b^	.	.	++++	++++	+
**mRNA degradation**					
*Degraded in early embryos:*					
De Renzis *et al.*, 2007 [58]^b^	.	.	++++	++++	.
Thomsen *et al.*, 2010 [59]^b^	+++	.	+++++	+++++	+++
Fly-FISH database ^c^	+	+	+++	+++	.
Degraded from 0 to 2 h to 2 to 4 h (modENCODE RNAseq)^a^	.	.	+++++	+++++	.
*Degraded via maternal degradation machinery*					
Tadros *et al.*, 2007 [60]^b^	.	.	++++	++++	.
Thomsen *et al.*, 2010 [59]^b^	.	.	+++	+++	.
*Degraded via zygotic degradation machinery*					
Thomsen *et al.*, 2010 [59]^b^	+++	+	+++	+++	+++

+ *P* = 0.01 to 0.05; ++ *P* = 0.001 to 0.01; +++ *P* = 10^−10^ to 0.001; ++++ *P* = 10^−10^ to 10^−30^; +++++ *P* <10^−30^; ‘.’ = not significant.

^a^ *P* values are Wilcoxon Rank Sum test. Also see Figure 6.

^b^ *P* values are Benjamini-Hochberg corrected Fisher’s exact test (that is, FDR). For complete details of comparisons see Additional file 10.

^c^ *P* values are Benjamini-Hochberg corrected Fisher’s exact test (that is, FDR). For details of comparisons see Additional file 9.

The subsets of transcripts bound only by BRAT or only by PUM had significantly lower translation indices as defined by Chen *et al.* [44] than the set of all expressed transcripts (Figure 6A; Table 3). Transcripts associated only with BRAT significantly overlapped with Qin *et al.*’s [57] translationally inactive transcripts and were depleted of translationally active transcripts (Table 3; Additional file 10). These results suggest that BRAT and PUM are able to mediate translational repression independent of one another. Not surprisingly, BRAT and PUM co-targets were also significantly correlated with translational repression by comparison to both datasets [44,57] (Figure 6A; Table 3; Additional file 10).

We next asked whether we could detect any relationship between PUM or BRAT binding and mRNA stability during the MZT in early embryos. For this analysis, we made use of a number of previously published datasets: from De Renzis *et al.* [58] and Thomsen *et al.* [59], which defined the entire set of maternal transcripts that are degraded during the MZT; from Fly-FISH [55,56], which annotates transcripts that are degraded at specific developmental stages; from modENCODE RNAseq, which measured transcript levels in embryos at various developmental stages, including embryos collected 0 to 2 h versus 2 to 4 h post egg-laying; and from Thomsen *et al.* [59] and Tadros *et al.* [60], which defined whether transcripts that are degraded during the MZT are cleared by maternally encoded (‘early’) and/or zygotically encoded (‘late’) decay machineries.

Our list of PUM-associated mRNAs was significantly enriched for unstable maternal mRNAs as defined by Thomsen *et al.* [59] (Table 3; Additional file 10). In addition, PUM-associated mRNAs were significantly enriched for transcripts annotated as ‘Degraded completely stage 5’ in the Fly-FISH database (Table 3; Additional file 9). Our PUM-associated transcripts were not enriched for ones eliminated by the early decay machinery, as defined in [59,60] (Table 3; Additional file 10). In contrast, our sets of PUM-associated mRNAs, of PUM-only targets, and of PUM-BRAT co-targets were significantly enriched for transcripts degraded by the late decay machinery (as defined by [59]). Moreover, the PUM-only transcripts were significantly enriched for ones annotated as ‘Degraded completely stage 5’ in the Fly-FISH database (Table 3; Additional files 9 and 10). These results are consistent with Thomsen *et al.*’s analysis of mRNAs associated with transgenically expressed PUM-RBD [37], which led them to conclude that PUM-RBD-bound transcripts undergo late decay. Taken together, our data are consistent with the hypothesis that PUM plays a role in mRNA degradation late during the MZT [58,59].

While PUM has previously been predicted to be involved in mRNA decay [59], there has heretofore been no evidence of a role for BRAT in mRNA destabilization. Strikingly, however, BRAT-associated mRNAs were very highly enriched for maternal transcripts that are degraded during the MZT as defined in De Renzis *et al.* [58] and Thomsen *et al.* [59] (Table 3; Additional file 10), as well as transcripts degraded during stages 4 to 5 as defined by the Fly-FISH database (Table 3; Additional file 9). Moreover, analysis of the modENCODE RNAseq data revealed that, for BRAT targets, the amount of RNA present in 2 to 4 h versus 0 to 2 h embryos is significantly lower than for the entire set of genes assayed in our RIP-Chip data set (Figure 6B; Table 3). Unlike PUM, BRAT targets were enriched for mRNAs degraded by both the early decay machinery as defined in both Thomsen *et al.* [59] and Tadros *et al.* [60], and the late decay machinery [59] (Table 3; Additional file 10). Notably, all of these enrichments were also true for the BRAT-only subset of mRNAs (Figure 6B; Table 3; Additional files 9 and 10).

Taken together, these data strongly suggest that, in addition to its role as a translational repressor, BRAT has an important PUM-independent function in mRNA destabilization during the MZT, acting as a component of both the early and the late decay machineries. Further supporting a role for BRAT in decay is the previously reported enrichment of a UUGUU motif among unstable maternal mRNAs [58], which carries the UGUU core of our BRAT consensus motif. Combined with the strong enrichment for unstable transcripts among our BRAT-associated mRNAs, these results lead to the prediction that BRAT is a regulator of transcript degradation during the MZT.

### BRAT reduces mRNA translation and stability in S2 tissue culture cells

As a first step towards assessing the possible role of BRAT in mRNA decay, we used the S2 tissue culture cell luciferase reporter assay described above (Figure 5A). RT-qPCR showed that the steady-state level of transcripts carrying the array of six wild-type BRAT-binding sites was lower than that of transcripts carrying the array of six point-mutated BRAT-binding sites (Figure 5D, Control RNAi), consistent with a role for BRAT in promoting mRNA degradation. This effect on the reporter mRNA carrying the wild-type BRAT-binding sites was mediated by BRAT, since reporter transcript levels increased more than two-fold upon RNAi-mediated BRAT knockdown (Figure 5D, *brat* RNAi 1 and 2). Comparison of the data in Figures 5C and D suggests that BRAT also translationally represses its targets as, upon BRAT knockdown, there was a greater increase in luciferase enzyme activity (approximately three-fold) than in luciferase mRNA levels (approximately two-fold). These results are consistent with a role for BRAT in both translationally repressing and inducing the degradation of its targets.

### BRAT-associated mRNAs are stabilized in embryos from *brat*-mutant females

**Figure 7:**
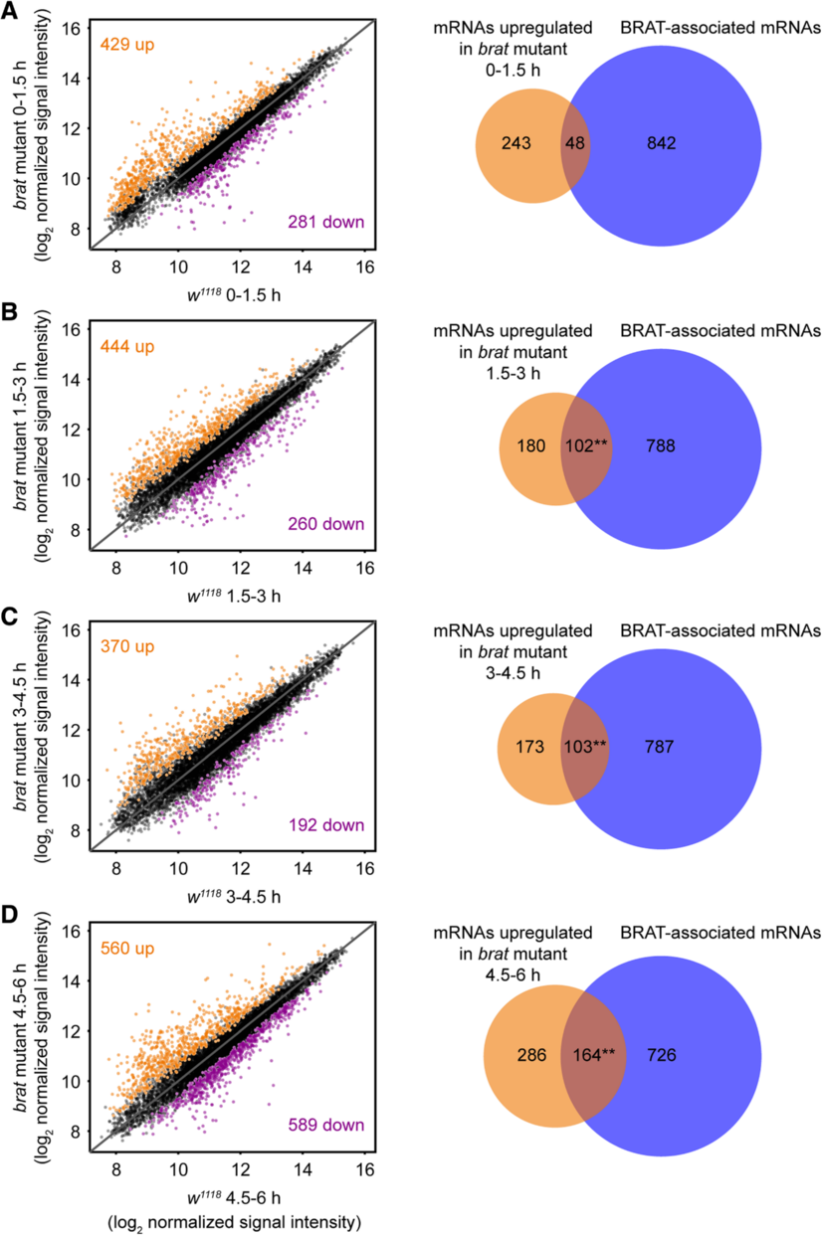
BRAT-associated mRNAs are globally upregulated in *brat*-mutant embryos. Scatterplots of RMA-normalized signal intensity in *brat* mutant versus *w* ^*1118*^ embryos at **(A)** 0 to 1.5 h, **(B)** 1.5 to 3.0 h, **(C)** 3.0 to 4.5 h, and **(D)** 4.5 to 6.0 h post egglaying. Plots show all transcripts represented on the microarray that were defined as expressed (see Methods). Values represent averages from three independent biological replicates. The solid diagonal line represents no enrichment. Transcripts up or down in *brat* mutant embryos compared to wild-type embryos with an FDR <5% were defined as up- or downregulated transcripts, and are highlighted with orange and magenta, respectively. Beside each plot are Venn diagrams depicting the overlap between transcripts upregulated at the corresponding timepoint and the set of BRAT-associated mRNAs defined by our RIP-Chip (also see Table 4). Note that these comparisons suggest that the transcripts upregulated at 1.5 to 3.0, 3.0 to 4.5, and 4.5 to 6.0 h are directly regulated by BRAT as they are enriched for BRAT-associated mRNAs. In contrast, those upregulated at 0 to 1.5 h likely reflect changes resulting from secondary effects of the *brat* mutation. The total number of BRAT-associated transcripts (890) shown in this figure and Figure 8 differs from the number in Figure 1 (1,197) because of differences in the set of expressed genes defined for these two experiments. We only considered the subset of BRAT targets identified in Figure 1 that were also defined as expressed in the *brat*-mutant time-course shown here. Likewise, in the Venn diagrams in this figure and in Figure 8 the numbers of transcripts upregulated in *brat* mutants differ from the numbers in the associated plots. **P <10^−11^ (Bonferroni-corrected Fisher’s exact test).

In light of the predicted novel function of BRAT in mRNA decay during the MZT we next used microarrays to globally compare the transcriptome of 0- to 1.5-, 1.5- to 3-, 3- to 4.5-, and 4.5- to 6-h-old embryos from *brat*-mutant females (referred to henceforth as ‘*brat* mutant embryos’) to wild-type embryos at the same stages. This resulted in the identification of 1,182 genes whose transcript levels were significantly higher and 1,022 genes whose transcript levels were significantly lower in *brat* mutants than wild type at one or more time points (FDR <5%; Figure 7A to D; Additional file 11).

If BRAT has a role in mRNA decay, one would expect that the genes that are upregulated in *brat* mutant embryos would be direct targets of BRAT. Indeed, there was a highly significant overlap between BRAT-associated mRNAs as defined by our RIP-Chip experiment and transcripts upregulated in *brat* mutant embryos at 1.5 to 3.0, 3.0 to 4.5, and 4.5 to 6.0 h (Figure 7B to D, Table 4, Bonferroni-corrected Fisher’s exact test *P* values ranged from 6.6 × 10^−12^ to 5.7 × 10^−20^). Moreover, the 3′UTRs of the upregulated transcripts were significantly enriched for BRAT-binding sites relative to co-expressed transcripts whose expression level was unchanged in *brat*-mutant embryos (Table 4, AUROC values ranged from 0.56 to 0.59; Bonferroni-corrected WMW Rank Sum *P* values ranged from 0.016 to 6.1 × 10^−6^; Additional file 12). Taken together, these data strongly suggest that the upregulation of transcripts at 1.5 to 3.0, 3.0 to 4.5, and 4.5 to 6.0 h is a direct consequence of the absence of BRAT binding.

**Table 4:** **Summary of comparisons to BRAT-associated mRNAs and of BRAT-binding motif enrichment tests for transcripts up- or downregulated in** ***brat*** **mutant embryos**

	**Upregulated in** ***brat*** **mutant embryos**				**Downregulated in** ***brat*** **mutant embryos**			
	**0-1.5 h**	**1.5-3 h**	**3-4.5 h**	**4.5-6 h**	**0-1.5 h**	**1.5-3 h**	**3-4.5 h**	**4.5-6 h**
Overlap with BRAT-associated mRNAs^a^	.	++++	++++	++++	.	.	- - -	- - -
Enrichment of BRAT-binding sites in 3’UTR^b^	.	+++	+++	+	.	- - -	- - -	- - - -

(+) or (−) *P* = 0.01 to 0.05; (++) or (− −) *P* = 0.001 to 0.01; (+++) or (− − −) *P* = 10^−10^ to 0.001; (++++) or (− − − −) *P* = 10^−10^ to 10^−22^; (+) symbols indicate significant overlap or enrichment, (−) symbols indicate significant non-overlap or depletion; ‘.’ = not significant (that is, *P* >0.05).

^a^ *P* values are Bonferroni-corrected Fisher’s exact test. For complete details of comparisons see Additional file 12.

^b^ *P* values are Bonferroni-corrected WMW *P* values. For complete details of comparisons, including AUROCs, see Additional file 12.

In contrast, at the 0- to 1.5-h time point, there was no significant overlap between the BRAT-associated mRNAs and those upregulated in *brat* mutant embryos (Figure 7A, Table 4, Bonferroni-corrected Fisher’s exact test *P* = 1) and the 3′UTRs of these transcripts were not enriched for BRAT-binding sites (AUROC = 0.52; Bonferroni-corrected WMW Rank Sum *P* = 1; Table 4; Additional file 12), suggesting that the upregulation of these transcripts at the first time point is not a direct result of BRAT binding and may, instead, be due to secondary effects associated with the *brat* mutant phenotype. Our analysis of the transcripts that were downregulated in *brat* mutant embryos also suggested they are not direct BRAT targets (Table 4, Additional file 12).

### BRAT promotes degradation of maternal mRNAs via both the early and late decay machineries

**Figure 8:**
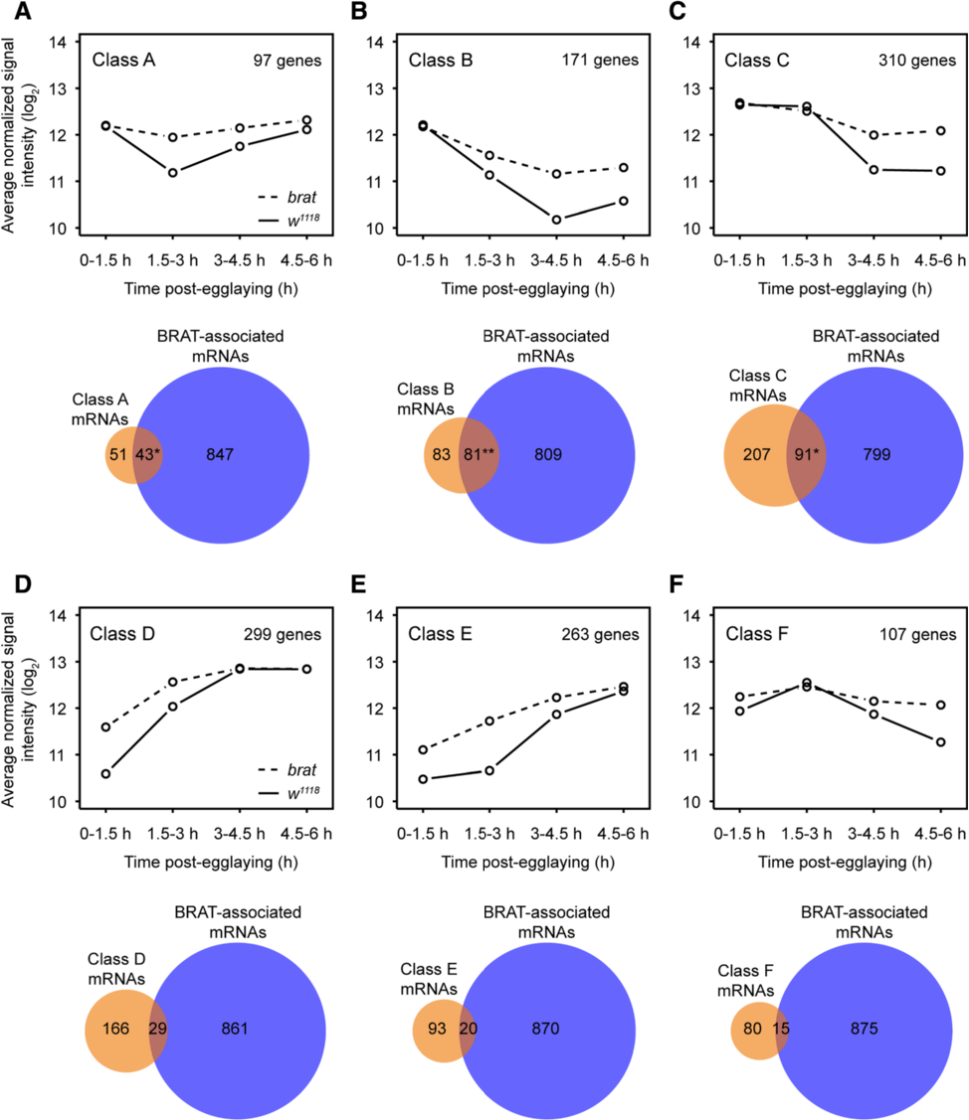
BRAT promotes decay of maternally expressed mRNAs during the MZT via both the early and late decay machineries. K-means clustering analysis of the union of transcripts upregulated at any time point in *brat* mutant embryos partitioned transcripts into six classes on the basis of their expression in wild-type embryos. **(A-F)** Plots showing the average expression levels (RMA-normalized signal intensity) of the transcripts in each class, in wild-type (solid line) and *brat* mutant (dashed line) embryos, from 0 to 6 h post egg-laying. Below each plot are Venn diagrams depicting the overlap of the transcripts in each class with the set of BRAT-associated mRNAs defined by our RIP-Chip. These comparisons indicate that Classes A, B and C are significantly enriched for BRAT-associated mRNAs and therefore are likely to be direct targets of BRAT. In contrast, Classes D, E and F show no enrichment for BRAT-associated mRNAs, and are therefore likely upregulated as a result of secondary effects of the *brat* mutation. Given that Classes A, B, and C comprise maternally-expressed transcripts that undergo decay during the MZT, via the early (Classes A and B) and late (Class C) machineries (see text and Table 5), their stabilization in *brat* mutants indicates a role for BRAT in decay of maternal mRNAs during the MZT through both the early and late decay machineries. *P <10^−6^, **P <10^−18^ (Fisher’s exact test).

Having demonstrated that BRAT-associated mRNAs are upregulated in *brat* mutant embryos, we next assessed the expression dynamics of the upregulated genes using k-means clustering [61,62]. This method subdivided the upregulated transcripts into six distinct classes based solely on their expression in wild-type embryos (Figure 8, Additional files 13 and 14). These six classes can be loosely divided into two subsets. The first subset includes Classes A, B, and C, which consist of transcripts that start out at relatively high levels at the 0- to 1.5-h time point and are degraded subsequently (Figure 8A to C; Additional file 14), suggesting that these classes largely comprise maternally expressed transcripts that are degraded during the MZT. Indeed, comparison of Class A, B, and C transcripts to previously published datasets [58-60] revealed that virtually all are maternally deposited and that Classes B and C are significantly depleted of zygotically transcribed mRNAs, while Class A appears to represent maternally-expressed mRNAs that are degraded and then re-expressed upon zygotic genome activation (Table 5; Additional file 15). The second subset includes Classes D, E, and F, which consist of transcripts that increase in abundance between the 0- to 1.5-h and the 1.5- to 3.0-h time points (Figure 8D to F; Additional file 14), suggesting that they primarily comprise transcripts that are expressed upon zygotic genome activation. Consistent with this, they are highly enriched for zygotically expressed transcripts, and Classes D and E are significantly depleted of maternally expressed transcripts (Table 5; Additional file 15).

**Table 5:** **Summary of comparisons of the six classes of transcripts upregulated in** ***brat*** **mutant embryos to other datasets** ^**a**^

	**Class A**	**Class B**	**Class C**	**Class D**	**Class E**	**Class F**
**BRAT-associated mRNAs**						
**(as defined in this study)**						
All BRAT targets	+++	++++	+++	.	.	.
BRAT-not-PUM targets	+++	++++	+++	- -	.	.
BRAT-and-PUM co-targets	.	+	+	+	.	.
**Maternally- vs. zygotically-expressed transcripts**						
*Maternally-expressed transcripts*						
Tadros *et al.*, 2007 [60]	.	.	+++	- - - -	- - - -	.
De Renzis *et al.*, 2007 [58]	.	++	+	- - - - -	- - - - -	.
Thomsen *et al.*, 2010 [59]	.	+++	+++	- - - - -	- - - - -	.
*Zygotically-expressed transcripts*						
De Renzis *et al.*, 2007 [58]	+++	- - -	- - - -	++++	+	.
Thomsen *et al.*, 2010 [59]	- - -	- - - -	- - - -	+++++	++++	++
**Maternally-expressed and degraded transcripts**						
*Maternally-expressed and degraded in early embryos*						
De Renzis *et al.*, 2007 [58]	++++	++++	- - -	- - - -	.	- - - -
Thomsen *et al.*, 2010 [59]	++++	++++	.	- - - -	.	- - - -
*Maternally-expressed and degraded in unfertilized eggs*						
Tadros *et al.*, 2007 [60]	+++	++++	- - -	- - - -	.	- - -
*Degraded exclusively via early decay machinery*						
Thomsen *et al.*, 2010 [59]	+	.	- -	- -	.	.
*Degraded via early and late decay machineries*						
Thomsen *et al.*, 2010 [59]	+++	++++	- - -	- - -	.	- - - -
*Degraded exclusively via late decay machinery*						
Thomsen *et al.*, 2010 [59]	.	+++	++++	- -	.	.
**Cooperation with other** ***trans*** **-acting factors in mRNA degradation**						
*Smaug-dependent unstable mRNAs*						
Tadros *et al.*, 2007 [60]	.	- - -	.	- - -	-	.
*miR-309 cluster-dependent unstable mRNAs*						
Bushati *et al.*, 2008 [65]	.	.	.	-	.	.
**Zelda transcriptional targets**						
*Bound by Zelda in promoter region by ChIP-Seq*						
Harrison *et al.*, 2011 [52]						
Nuclear cycle 8	.	.	.	++++	.	++
Nuclear cycle 13	.	.	.	++++	.	+
Late nuclear cycle 14	.	.	-	++++	+	.
*Downregulated in zelda mutant embryos*						
Liang *et al.*, 2008 [53]	.	.	.	++	.	+++

(+) or (−) *P* = 0.01 to 0.05; (++) or (− −) *P* = 0.001 to 0.01; (+++) or (− − −) *P* = 10^−10^ to 0.001; (++++) or (− − − −) *P* = 10^−10^ to 10^−30^; (+++++) or (− − − − −) *P* <10^−30^; (+) symbols indicate significant overlap or enrichment, (−) symbols indicate significant non-overlap or depletion; ‘.’ = not significant (that is, *P* >0.05). *P* values are Benjamini-Hochberg corrected Fisher’s exact test (that is, FDR). For complete details of comparisons see Additional file 15.

^a^We note that transcripts that were downregulated in *brat* mutant embryos at the 1.5- to 3.0-h and 4.5- to 6.0-h time points were also modestly enriched for mRNAs produced by direct targets of Zelda [52] (Benjamini-Hochberg corrected Fisher’s exact test *P* = 0.016 and 0.032, respectively) and those downregulated at 1.5 to 3.0 h were strongly enriched for genes downregulated in *zelda*-mutant embryos [53] (Benjamini-Hochberg corrected Fisher’s exact test *P* = 6.6 × 10^−9^). We speculate that increased Zelda expression in 0 to 1.5 h *brat*-mutant embryos disrupts the normal cascade of zygotic genome activation resulting in downregulation of a subset of Zelda’s targets at the later time points.

To understand the nature of BRAT’s potential role in their regulation, we assessed the enrichment of BRAT-associated mRNAs (as defined by our RIP-Chip experiment) in each expression class. Strikingly, this revealed that each of Classes A, B, and C were very significantly enriched for BRAT-associated mRNAs, suggesting that these mRNAs are direct BRAT targets (Table 5; Additional file 15). In contrast, Classes D, E, and F were not enriched for BRAT-associated mRNAs, suggesting that their upregulation is likely to result from indirect effects of the *brat* mutant phenotype (Table 5; Additional file 15). The segregation of BRAT-associated mRNAs into Classes A through C is especially impressive when one considers that the six classes were generated through analysis of the expression of these transcripts only in wild-type embryos. Together, these data strongly support a major role for BRAT in directly regulating the destabilization of its target maternal mRNAs during the MZT.

A closer examination of the expression patterns of the transcripts present in Classes A through C suggests that BRAT functions in both early and late decay pathways, as our earlier analysis of BRAT-associated mRNAs had predicted. In wild-type embryos, Class C transcripts remained stable through the first two time points, but were degraded by the 3.0- to 4.5-h time point, indicating decay through a late pathway. In contrast, Class A and B mRNAs decreased between the 0- to 1.5-h and the 1.5- to 3.0-h time points, suggesting decay through an early pathway. Whether Class A and B mRNAs are also degraded via late pathways is unclear. The rate of decrease of mRNAs in Class B was constant between the 0- to 1.5-h and 3.0- to 4.5-h time points suggesting that they are not also attacked by a late-acting machinery; however it remains possible that a maternal machinery acting on these mRNAs is replaced by one that acts zygotically. The expression of Class A mRNAs increased in wild type at the 3.0- to 4.5-hour time point, indicating that these genes are also zygotically transcribed; this prevented us from determining whether Class A mRNAs are also subject to degradation via a late pathway. We note that Class A transcripts significantly overlap with those previously defined [59] as degraded via ‘maternal only’ and ‘maternal plus zygotic’ pathways; Class B transcripts with ‘maternal plus zygotic’ and ‘zygotic only’ pathways; and Class C transcripts with ‘zygotic only’ pathways (Table 5). These overlaps are consistent with our assessment of the expression patterns of these different classes, and support the conclusion that BRAT promotes mRNA degradation via both early and late decay pathways.

### Maternally loaded *hb* mRNA is stabilized in embryos lacking functional BRAT protein

**Figure 9:**
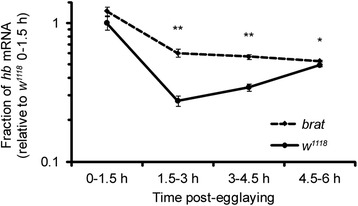
Maternally expressed *hb* mRNA is stabilized in *brat*-mutant embryos. Levels of the maternally expressed isoform of *hb* mRNA (*hb*-RB) were assayed by RT-qPCR in *brat* mutant or wild-type embryos collected 0 to 1.5, 1.5 to 3.0, 3.0 to 4.5, and 4.5 to 6.0 h post egg-laying, and normalized to levels of *RpL32* mRNA, whose levels are stable throughout this time-course. *hb* mRNA levels were significantly higher in *brat* mutant than wild-type embryos at 1.5 to 3.0, 3.0 to 4.5, and 4.5 to 6.0 h (values represent average of three biological replicates +/− standard error of the mean). *P <0.05, **P <0.005 (Student’s t-test).

Prior to our transcriptome-wide analyses reported here, the *hb* mRNA was the only known direct target of BRAT, which was shown to repress a luciferase-*hb 3*′*UTR* reporter mRNA in S2 cells [17]. Our RIP-Chip experiments have shown that *hb* mRNA co-purifies with BRAT in early embryos. It therefore was of particular interest to ask whether BRAT is required for degradation of maternal *hb* transcripts, in addition to the well-established role for BRAT in translational repression of *hb* mRNA. Since *hb* transcripts are loaded maternally (the *hb-RB* mRNA isoform) as well as synthesized zygotically (the *hb-RA* mRNA isoform), and the probes on our microarray did not distinguish between these isoforms, we carried out RT-qPCR with maternal-*hb*-specific (*hb-RB*) primers to assess whether it was stabilized in embryos produced by *brat*-mutant females. Consistent with a role for BRAT in destabilizing the maternal transcripts, *hb-RB* was present at significantly elevated levels in *brat* mutant compared to wild-type embryos, particularly at the 1.5- to 3-h and 3- to 4.5-h time points (Figure 9). We conclude that BRAT mediates both translational repression and degradation of maternal *hb* mRNA.

### Analysis of the functions and localization patterns of mRNAs stabilized in *brat* mutants

We next asked whether any particular annotated functions or localization patterns were enriched among transcript Classes A, B, and C. Strikingly, the GO terms enriched in these three classes were nearly identical to those enriched among the entire set of BRAT-associated mRNAs and included ‘intrinsic to membrane’, ‘ion transport’, ‘phosphate metabolic process’, ‘Jak-STAT signaling’, ‘embryonic pattern specification’, and terms related to lipid and glycerophospholipid metabolism (Table 6; Additional file 16). A small number of GO terms, however, were enriched among the stabilized transcripts but not among the set of BRAT-associated mRNAs (for example, ‘starch and sucrose metabolism’). Localization patterns enriched among the stabilized mRNAs included posterior and pole-cell localization as well as perinuclear localization, again consistent with those we had found to be enriched among BRAT-associated mRNAs (Table 7; Additional file 17).

**Table 6:** **Comparison of gene annotation enrichment analyses for BRAT-associated mRNAs and mRNAs upregulated in** ***brat*** **mutant embryos in Classes A, B, and C**

	**All BRAT-associated mRNAs**	**mRNAs associated only with BRAT**	**PUM and BRAT co-target mRNAs**	**mRNAs upregulated in** ***brat*** **mutant embryos**		
				**Class A**	**Class B**	**Class C**
**Biological and developmental**						
Embryonic pattern specification/Axis specification	+	.	+	.	.	+
Neuroblast fate determination	.	.	+	.	.	.
**Cellular**						
Cell motility/cell migration	.	.	+	.	.	.
**Molecular**						
Signal transduction	+	.	+++	.	.	+
Regulation of transcription	.	.	+	.	.	.
Protein kinase activity	+	.	.	.	.	+
Phosphatase activity	+	.	.	.	.	+
Integral membrane proteins	+++++	+++	+++	.	+	.
Ion transport/homeostasis	+++	++	+	.	+++	.
Phospholipid/phosphoinositide metabolism	+++	+++	.	.	+	.

+ FDR = 1% to 10%; ++ FDR = 0.1% to 1%; +++ FDR = 0.001% to 0.1%; ++++ FDR = 10^−5^% to 0.001%; +++++ FDR <10^−5^%; ‘.’ = not significant (FDR >10% and Benjamini *P* value >0.1). FDR values represent the most significantly enriched annotation term related to the function listed. Analyses were performed using the DAVID functional annotation tool [42,43]. For details see Additional files 8 and 16.

**Table 7:** **Comparison of Fly-FISH localization patterns enriched among BRAT-associated mRNAs and mRNAs upregulated in** ***brat*** **mutant embryos in Classes A, B, and C**

	**All BRAT-associated mRNAs**	**mRNAs associated only with BRAT**	**PUM and BRAT co-target mRNAs**	**mRNAs upregulated in** ***brat*** **mutant embryos**		
				**Class A**	**Class B**	**Class C**
**Embryonic stages 1 to 3 localization patterns**						
Posterior localization	++	.	++	.	.	.
Nuclei-associated/Perinuclear	++	.	++	.	.	++++
**Embryonic stages 4 to 5 localization patterns**						
Patterns indicative of zygotic transcription	.	.	+	.	.	.
Pole cell enrichment	+	.	.	.	+++	+
Yolk plasm localization	.	+	.	.	.	.

+ FDR = 1% to 10%; ++ FDR = 0.1% to 1%; +++ FDR = 0.001% to 0.1%; ++++ FDR = 10^−5^% to 0.001%; +++++ FDR <10^−5^%; ‘.’ = not significant (FDR >10%). FDR values indicated represent the most significantly enriched term related to the localization pattern listed. For details see Additional files 9 and 17.

The fact that the annotated functions and localization patterns enriched among Classes A, B, and C so closely matched those enriched among the set of BRAT-associated mRNAs further supports our conclusion that these three classes of transcripts largely represent direct targets of BRAT.

### Expression of Zelda (Vielfaltig) target genes is misregulated at early time points in *brat* mutant embryos

As described above, Classes D through F largely comprise zygotically expressed transcripts that are upregulated in *brat* mutants in a manner not directly dependent on BRAT. This led us to ask whether misexpression of any of the transcription factor mRNAs bound by BRAT could explain their upregulation. The mRNA encoding Zelda (VFL), which is essential for normal zygotic genome activation [52-54], was strongly associated with BRAT in our RIP-Chip experiments (5.1-fold enriched, FDR = 0%). We, therefore, asked whether any of the upregulated transcripts in Classes D, E, and F correspond to genes previously defined as Zelda (VFL) targets. Strikingly, all three classes were significantly enriched for transcripts encoded by genes whose promoters have been shown to be directly bound by Zelda (VFL) as assayed via ChIP-Seq [52] (Table 5; Additional file 15). There was a similar enrichment among Classes D and F, but not Class E, for transcripts previously shown to be downregulated in *zelda (vfl)* mutant embryos [53] (Table 5; Additional file 15).

In *brat*-mutant embryos transcripts in Classes D, E, and F are upregulated at the 0- to 1.5-h time point, which overlaps with the phase where Zelda/VFL is known to bind its target genes and activate their transcription [52-54]. We therefore hypothesize that, in wild-type embryos, the BRAT RBP represses the *zelda (vfl)* mRNA. In *brat* mutant embryos, then, Zelda (VFL) would be upregulated, leading to upregulation of its target genes (for example, *hb* [54]; Additional file 18 shows the effect on zygotically transcribed *hb* in *brat* mutants). Consistent with this model is the fact that increasing the number of Zelda (VFL) binding sites in a Zelda (VFL) target gene results in earlier transcription of that gene [63,64]. In addition, while the *zelda* (*vfl*) mRNA is bound by BRAT, we did not detect a change in *zelda* (*vfl*) mRNA levels in *brat* mutant embryos. Thus, BRAT is likely to regulate the *zelda* (*vfl*) mRNA at the level of translation. However, with respect to upregulation of Zelda’s target genes, we cannot exclude the possibility that BRAT also regulates either the stability or the translation of mRNAs encoding co-factors of Zelda.

### The role of other *trans*-acting factors in BRAT-dependent mRNA decay

SMG has previously been identified as a major regulator of maternal mRNA elimination via a maternally encoded, early decay pathway in the embryo [60] while the *miR-309* cluster of miRNAs, which is expressed zygotically, has been shown to act via a late pathway [65,66]. Comparisons of the mRNAs in Classes A, B, and C with mRNAs that require either SMG or the *miR-309* cluster for their degradation showed no significant overlap (Table 5; Additional file 15), suggesting that BRAT does not cooperate with SMG or the *miR-309* miRNAs in transcript degradation. BRAT-mediated mRNA degradation may therefore represent a novel pathway for both early and late transcript decay during the MZT. BRAT has previously been found to co-immunoprecipitate with the NOT1 subunit of the CCR4/NOT deadenylase complex [67], suggesting that BRAT, like SMG [68], may induce transcript decay through a mechanism that involves deadenylase recruitment to target transcripts.

PUM-RBD-bound mRNAs are enriched in those degraded late [59] and we have shown here that PUM-associated mRNAs immunoprecipitated with our synthetic anti-PUM Fab are enriched for maternal transcripts that undergo late decay. Since a subset of BRAT-associated mRNAs also undergoes late decay, our data suggest that BRAT-mediated late decay could involve cooperation with PUM. As discussed earlier, Class C BRAT-dependent mRNAs are unambiguously degraded through a late pathway. Interestingly, we observed a significant overlap of Class C mRNAs with those co-associated with both PUM and BRAT (but not with PUM-only targets) (Table 5; Additional file 15), suggesting that PUM and BRAT are likely to cooperate in late decay of their shared target transcripts.

## Conclusions

Here we have shown that PUM and BRAT each bind to hundreds of mRNAs in early *Drosophila* embryos. Our data suggest that BRAT and PUM recognize a relatively small proportion of co-targets whereas the majority of targets are bound only by BRAT or only by PUM. Thus, BRAT and PUM function largely independently. Through computational analysis of PUM- and of BRAT-associated transcripts, we have identified the previously known PUM motif and a novel consensus motif for BRAT binding *in vivo* that is distinct from the PUM motif*.* The motif we identified for *in vivo*-bound transcripts is almost identical to one that we identified *in vitro* using RNAcompete. This represents the first RNA-binding motif identified for a TRIM-NHL protein. We verified the functionality of this motif by demonstrating that it confers BRAT-dependent repression on luciferase reporter mRNAs in S2 tissue culture cells.

Analysis of the functions of PUM- and BRAT-associated mRNAs identified a variety of novel roles for the post-transcriptional regulatory activity of these RBPs in early embryos. For PUM, these include a wider than previously appreciated role in embryonic pattern formation as well as a role in cell motility and post-transcriptional regulation of mRNAs encoding transcription factors, signal transduction components and membrane proteins. In contrast, BRAT plays a role largely in the post-transcriptional regulation of mRNAs encoding membrane proteins and proteins functioning in membrane-related processes.

Both PUM- and BRAT-associated mRNAs are translated at low levels, consistent with their previously known roles as translational repressors. We also assessed a potential role for PUM and BRAT in regulating mRNA stability during the MZT. While analysis of the identified target RNAs suggests that PUM’s role is primarily in late, zygotically encoded decay, BRAT is predicted to function in both early and late decay. Consistent with these correlations we have shown that BRAT-mediated repression in *Drosophila* tissue culture cells occurs at the level of both translational repression and mRNA destabilization. A global role for BRAT in mRNA decay during the MZT was demonstrated by time-course microarray analysis of *brat*-mutant embryos in which hundreds of BRAT targets were stabilized. These data are consistent with BRAT being an important component of both early and late decay machineries during the *Drosophila* MZT, and representing a novel pathway for both early and late mRNA decay.

## Methods

### *Drosophila* stocks

*Drosophila* stocks used were as follows: *w*^*1118*^, *brat*^*fs1*^/*CyO* [69], and *Df(2L)TE37C-7*/*CyO* [70]. The *brat*^*fs1*^ mutation results in a G774D amino acid substitution in the NHL domain that causes female sterility [3,27,69]. The G774D mutation causes defects in RNA-binding [17].

### Generation and purification of anti-BRAT and anti-PUM synthetic antibodies

Synthetic antibodies were generated against antigens comprising BRAT amino acids 375–565 (numbering according to BRAT-PA isoform), and PUM amino acids 726–882 (numbering according to PUM-PA isoform), each expressed and purified from *E. coli* as GST fusion proteins, as described [32]. Five rounds of binding selection with synthetic antibody Library F [71] were performed against GST-BRAT^375–565^ and GST-PUM^726–882^ coated on 96-well Maxisorp Immunoplates (NUNC, Rochester, NY, USA) as described [72]. Each round included negative selection to remove GST binders from the phage pool, through pre-incubation of phage with purified GST, either immobilized on 96-well Maxisorp Immunoplates by coating overnight at 4°C at a concentration of 5 μg/mL (rounds 1 to 2), or in solution at a concentration of 0.5 mg/mL (rounds 3 to 5). After five rounds of selection, phage were produced from individual clones and used in phage ELISAs to identify clones that bound the RBP antigen but not GST, as described [72]. Such clones were submitted to DNA sequencing to identify unique Fabs.

The two synthetic antibodies used in this study, anti-BratA1 Fab and anti-Pum7 Fab, were then expressed and purified from *E. coli* as FLAG- and 6xHis-tagged Fabs, as described [32].

### Immunoprecipitations

Extract was prepared from embryos collected 0 to 3 h post egg-laying. Embryos were disrupted in a minimal volume of lysis buffer: 150 mM KCl, 20 mM HEPES-KOH (pH 7.4), 1 mM MgCl_2_, 1 mM DTT, protease inhibitors (1 mM AEBSF, 2 mM benzamidine, 2 μg/mL pepstatin, 2 μg/mL leupeptin). After centrifugation, the supernatant was recovered and stored at −80°C.

For RIP-Chip experiments, 400 μL of embryo extract was supplemented with Triton X-100 to 0.1%, then diluted in an equal volume of lysis buffer with 0.1% Triton X-100. This diluted extract was re-centrifuged, and incubated for 3 to 4 h at 4°C with 40 μL of anti-FLAG M2 affinity gel (Sigma) carrying 20 μg of purified FLAG-tagged Fab and blocked with BSA. Beads were washed four times with cold wash buffer A (lysis buffer plus 0.1% Triton X-100), and three times with cold wash buffer B (100 mM NaCl, 100 mM HEPES-NaOH (pH 7.4), 1 mM MgCl_2_). Fabs and associated protein and RNA were then eluted from the beads by one 20 min elution at 4°C with 100 μL of FLAG-peptide (Sigma; 200 μg/mL in wash buffer B), and RNA was isolated from the eluate using TRI Reagent (Sigma).

For immunoprecipitations for western blot, immunoprecipitations were carried out as above but at a smaller scale, using 50 μL of initial embryo extract, 5 μL of anti-FLAG M2 affinity gel carrying 2.5 μg of purified FLAG-tagged Fab, and eluting with 25 μL of FLAG peptide. SDS-PAGE sample buffer was then added to eluate for western blot analysis (see Additional file 1).

### Microarray analysis of BRAT and PUM RIP samples

For microarray analysis of RIP samples, double-stranded cDNA was prepared following the protocol described in the NimbleGen Array User’s Guide (Gene Expression Arrays, version 6.0), but using a primer mixture containing 50 ng/μL random hexamer primers and 67 pmol/μL anchored-oligo-dT primers. Specifically, double-stranded cDNA was prepared from 150 ng or 200 ng of immunoprecipitated RNA. 400 or 500 ng of double-stranded cDNA was labelled with Cy3- or Cy5-tagged random nonamers following the Roche NimbleGen protocol. Labelled cDNA was then hybridized to custom-designed *Drosophila* 4 × 72 K NimbleGen arrays (GEO platform number: GPL10539): each array was hybridized with 1 μg each of one Cy3- and one Cy5-labelled IP-RNA-derived sample, and washed, according to the NimbleGen protocol. Three biological replicates each were performed for anti-BRAT, anti-PUM, and control immunoprecipitated samples. Arrays were scanned with a GenePix4000B microarray scanner system (Molecular Devices, Inc., Sunnyvale, CA, USA). Scanned images were initially quantified using Nimblescan (Roche) and the resulting data were normalized using the ArrayStar 3 (DNASTAR) software using the RMA quantile method. BRAT and PUM immunoprecipitation samples were each normalized along with the control, separately.

To identify BRAT- or PUM-associated mRNAs, microarray data were analyzed using the Significance Analysis of Microarrays (SAM) [73] function available in the MultiExperiment Viewer software application [61,62]. Prior to performing SAM analysis, data from the immunoprecipitation samples were filtered such that only the subset of transcripts which we previously defined as expressed in 0- to 3-h embryos [33] was analyzed. Note that total RNA isolated from input embryo extract for the BRAT and PUM RIPs was also analyzed by microarray and determined to be very highly correlated with this previous analysis of transcript expression in 0- to 3-h embryos (data not shown). The normalized microarray signal intensities in the anti-BRAT or anti-PUM immunoprecipitations were then compared to the normalized microarray signal intensities in the control immunoprecipitations for all transcripts in the expressed set, using SAM two-class paired analysis. Genes significantly enriched in the anti-BRAT or anti-PUM immunoprecipitates compared to the control immunoprecipitate, with an FDR of less than 5% and at least 1.5-fold enrichment, were defined as BRAT- or PUM-associated mRNAs. The co-expressed non-targets used in the motif discovery and analysis of translation index and RNAseq data were those that were defined as expressed but which were negatively enriched in the ‘two-class paired analysis’ (that is, enriched in the control RIPs) for either the anti-BRAT or anti-PUM RIPs.

For the purposes of all subsequent analyses, gene IDs were updated to the most recent version (FlyBase release 5.56) by taking the FlyBase FBgn IDs present in the array gene description file and updating them using the ‘Upload/Convert IDs’ tool available on FlyBase.

### Motif discovery

The BRAT and PUM motifs were identified using the #ATS model, as described [38]. Briefly, the #ATS model is a discriminative *de novo* motif discovery algorithm, which takes as input positive (that is, bound or target transcripts) and negative (that is, co-expressed unbound or non-target transcripts) gene lists, and outputs the RNA motif that best distinguishes the positive and negative transcripts by having more accessible motif matches among the bound transcripts. Initial motif discovery was carried out on the relevant entire gene list. Then, to assess the predictive power of the motif(s), we calculated AUROC scores using a 3 × 10 cross-validation procedure. Specifically, we randomly split the positive and negative sets into 10 equal size bins, trained the motif using 9 of 10 bins (that is, the training set) and evaluated the predictive power of the motif on the remaining bin (that is, the test set). We repeated this procedure three times to avoid potential bias in the splitting procedure, and finally collected 30 test set AUROCs.

Note that the AUROC is a non-parametric measure of the difference of medians between two distributions. Significance is assigned to an AUROC using a Wilcoxon-Mann–Whitney rank sum test. A perfect AUROC of 1.0 would indicate that motif scores of all targets are higher than scores of all co-expressed non-targets. Thus, as examples, an AUROC of 0.8 means that, on average, targets have motif scores that are higher than those of 80% of the co-expressed non-targets, indicating enrichment of the motif; an AUROC of 0.5 means that, on average, targets have motif scores that are no different from those of co-expressed non-targets; and an AUROC of 0.4 means that, on average, targets have motif scores that are higher than 40% (that is, lower than 60%) of the co-expressed non-targets, indicating depletion of the motif.

### Motif enrichment tests on target transcripts and transcripts with altered levels in *brat* mutants

To evaluate the enrichment of a motif in the positive set versus the negative set, we assigned each transcript a score equal to the sum of the accessibilities of the motif matches in the transcript, and then used the AUROC score to evaluate how well that score distinguished bound and co-expressed unbound transcripts. The transcripts without a motif match were assigned a score of zero. The same strategy was used to evaluate motif enrichment (AUROC >0.5) or depletion (AUROC <0.5) in transcripts whose expression changed significantly in *brat*-mutant embryos relative to wild-type embryos, using co-expressed transcripts whose levels were unchanged as the control set (see below).

### Source of transcript sequences for motif finding

The *Drosophila melanogaster* (BDGP5.4) transcript sequences were downloaded from Ensembl using BioMart [74]. When there were multiple isoforms for a gene, we used the longest isoform to represent the gene.

### Quantifying target site accessibility for motif finding

Target site accessibility was assessed using RNAplfold. We fixed W = 80 and L = 40 and set U to the width of the motif, as previously described [38]. When calculating target site accessibilities for a 5′UTR, ORF, or 3′UTR site, we input the entire transcript into RNAplfold to ensure that the target site accessibility for sites immediately around the start codon or the stop codon was calculated correctly.

### Analysis of BRAT RNA-binding using RNAcompete

The RNA pool generation, RNAcompete pulldown assays, and microarray hybridizations were performed as previously described [39,41]. Briefly, GST-tagged BRAT NHL domain (amino acids 756–1037 of *Drosophila* BRAT isoform A as defined by Flybase) (20 pmoles) and RNA pool (1.5 nmoles) were incubated in 1 mL of Binding Buffer (20 mM HEPES pH 7.8, 80 mM KCl, 20 mM NaCl, 10% glycerol, 2 mM DTT, 0.1 μg/μL BSA) containing 20 μL glutathione sepharose 4B (GE Healthcare) beads (pre-washed 3 times in Binding Buffer) for 30 min at 4°C, and subsequently washed four times for 2 min with Binding Buffer at 4°C. 7-mer Z-scores and motifs were calculated as described previously [39].

### Data access

The BRAT and PUM RIP-Chip microarray data reported in this study have been deposited in NCBI’s Gene Expression Omnibus and are accessible through GEO series accession number GSE60466. The BRAT RNAcompete data are accessible at GEO Accession Number GSE60498 and Agilent AMADID number 024519. The *brat* mutant time-course microarray data are accessible through GEO series accession number GSE65661.

### Gene annotation enrichment analysis

The DAVID functional annotation tool web server [42,43] was used to perform enrichment analysis for GO terms included in the GO FAT database, as well as for terms included in a number of additional databases: COG ontology, Swissprot keywords, Uniprot sequence features, KEGG pathways, PIR Superfamilies, Interpro domains, and SMART domains, as per the default settings of the DAVID functional annotation tool. Genes identified as encoding BRAT- or PUM-associated mRNAs were analyzed for enrichment against the background of expressed genes defined for our RIP-Chip experiments, described above. Genes identified as upregulated in *brat* mutant embryos, in Classes A, B, or C, were analyzed for enrichment against the background of expressed genes defined in the *brat*-mutant time-course microarray experiment (see below). Terms or features enriched at an FDR of less than 10% and/or a Benjamini *P* value of less than 0.1 were considered significant.

### Localization pattern enrichment analysis

The subcellular localization, as annotated in the Fly-FISH database [56], of BRAT- and PUM-associated transcripts, as well as transcripts upregulated in *brat* mutant embryos, was analyzed to ask whether these transcripts were enriched for particular localization patterns (Fly-FISH annotations analyzed were up to date as of March 2014 for RIP-Chip data, and January 2015 for *brat* mutant microarray data). Analyses of localization patterns for embryonic stages 1 to 3 and embryonic stages 4 to 5 were carried out as described [33], with the following modifications: all localization terms present in the database were tested for enrichment, with the exception of the broadest terms at the top of the database hierarchy with no specific localization information, which were excluded from testing, as well as terms that did not contain more than 25 genes in the background set. Enrichment was determined using a two-sided Fisher’s exact test, (‘fisher.test’ function in R software with the alternative hypothesis = ‘two.sided’), and *P* values were adjusted for multiple comparisons using the Benjamini-Hochberg method to determine FDRs (using the ‘p.adjust’ function in R software with method = ‘fdr’). FDRs were calculated separately for each analysis of a single gene list versus a single Fly-FISH developmental stage. Localization terms enriched at an FDR of less than or equal to 10% were considered significant. Genes identified as encoding BRAT- or PUM-associated mRNAs were analyzed for enrichment against the background of expressed genes defined for our RIP-Chip experiments, described above. Genes identified as upregulated in *brat* mutant embryos, in Classes A, B, or C, were analyzed for enrichment against the background of expressed genes defined in the *brat* mutant time-course microarray experiment (see below).

### Comparisons of BRAT- and PUM-associated mRNAs to translation and stability datasets

For comparisons of BRAT- and PUM-associated mRNAs to previously published translation and stability datasets, datasets were obtained either from the publications themselves [57,59], or were provided to us by the authors [58]. For lists from Thomsen *et al.* [59], the entire set of maternally expressed unstable mRNAs in early embryos was considered as Classes II through V as defined in that publication; mRNAs degraded by the maternal, or early, pathway, were considered as Classes II and III (‘exclusively maternally degraded’ and ‘maternally degraded and transcribed’); and mRNAs degraded by the zygotic, or late, pathway, were considered as Class IV (‘exclusively zygotically degraded’). Prior to comparisons with PUM- and BRAT-associated mRNAs, all gene IDs were updated using the ‘Upload/Convert IDs’ tool available on FlyBase, to IDs consistent with FlyBase release 5.56. Enrichments and depletions for comparisons to data summarized in Additional file 10, from Qin *et al.* [57], De Renzis *et al.* [58], Thomsen *et al.* [59], Tadros *et al.* [60], were determined using a two-sided Fisher’s exact test, followed by multiple testing correction using the Benjamini-Hochberg procedure to determine FDRs (using R software). Multiple test corrections were carried out separately for comparisons of each list of target mRNAs to these datasets. Enrichments or depletions with an FDR less than 5% were considered significant.

For analysis of RNAseq data, total developmental RNAseq data were downloaded from FlyBase release 5.56, and the ratio of RNA (RPKM) in 2- to 4-h embryos versus 0- to 2-h embryos was calculated. Significant differences to the ratios for the set of all expressed genes defined by our microarray experiments were calculated using a two-sided Wilcoxon Rank Sum test.

For analysis of translation index data, translation indices in wild-type embryos were analyzed, and significant differences to the set of all expressed genes defined by our microarray experiments were calculated using a two-sided Wilcoxon Rank Sum test.

Analysis of degradation terms from the Fly-FISH database was carried out as part of the analysis of enrichment of localization terms from the Fly-FISH database, described above.

### *Drosophila* tissue culture and transient transfection

*Drosophila* S2 cells were maintained in SF 900 III SMF (Life Technologies) at 25°C. Luciferase reporters carry the firefly luciferase open reading frame in pRmHa3 [75] with either six wild-type or six point-mutated BRAT-binding sites inserted 59 nucleotides downstream of the luciferase open reading frame’s stop codon. The DNA sequence of the wild-type array of BRAT-binding sites is: TTGTTAT A TTGTTAT A TTGTTAT A TTGTTAT A TTGTTAT A TTGTTAT (‘TTGTTAT’ encodes the BRAT site and ‘A’ is a spacer), while in the mutated array all G residues were changed to C. A plasmid carrying the Renilla luciferase open reading frame inserted into pRmHa3 served as a transfection control. A mixture of 10 ng of firefly luciferase plasmid, 10 ng of Renilla luciferase plasmid, and 380 ng of pSP72 DNA (Promega) was transfected into 0.8 mL of cells at a density of 10^6^ cells/mL using 1.2 μL of X-tremeGENE 9 (Roche) according to the manufacturer’s instructions. Firefly and Renilla luciferase activities were measured approximately 48 h post transfection using the Dual-Luciferase Reporter Assay System (Promega) according to the manufacturer’s instructions. Note that, while expression of the luciferase open reading frames in pRmHa3 is under the control of the metal-inducible metallothionein promoter, we did not induce promoter expression but instead relied on basal-promoter activity to drive reporter-gene expression. In experiments involving RNAi knockdown, 0.8 mL of cells at a density of 10^6^ cells/mL were treated with 5 μg of double-stranded RNA generated via *in vitro* transcription using PCR products as templates. Double-stranded RNA was generated via *in vitro* transcription using T7 RNA polymerase. The DNA templates for *brat* knockdown were generated using primers as described by Weidmann and Goldstrohm [25], while control double-stranded RNA was transcribed from a PCR product corresponding to a portion of the Ampicillin-resistance gene and generated with the following primers: GGATCCTAATACGACTCACTATAGGGAAAGTTCTGCTATGTGGCGCGG and GGATCCTAATACGACTCACTATAGGGTCCTGCAACTTTATCCGC. Three days after the addition of double-stranded RNA, luciferase-reporter-encoding plasmids were transfected and their expression assayed as described above.

For analysis of luciferase reporter RNA levels, as well as quantitation of RNAi-mediated *brat* knockdown, RNAi knockdown and transfections were carried out as described, and approximately 48 h post-transfection cells were harvested and resuspended in TRI reagent (Molecular Research Center, Inc., Cincinnati, OH, USA), and total RNA was isolated following the manufacturer’s protocol. For each sample, 2 μg of total RNA were treated with DNase I (Invitrogen, catalog no. 18068–015) according to the manufacturer’s protocol. A total of 1 μg of DNase I-treated RNA was then used to prepare single-stranded cDNA by reverse transcription using random hexamer primers and Superscript II reverse transcriptase (Invitrogen), following the manufacturer’s instructions. The single-stranded cDNA was used to perform quantitative real-time PCR with primers specific to either the firefly luciferase ORF, the Renilla luciferase ORF, *brat*, or *RpL32*, using SYBR green PCR master mix (ABI) and a CFX384 Real-Time System (Bio-Rad). Relative levels of the various transcripts were determined using a standard curve. In parallel to the reverse transcription, the second 1 μg of DNase I-treated RNA was used in a control no-reverse transcriptase (no-RT) reaction, which was also subjected to qPCR and confirmed the measured signal was not a result of amplification of plasmid or genomic DNA.

### Microarray analysis of *brat* and *w*^*1118*^ embryo time-course samples

To compare the transcriptome of wild-type and *brat* mutant embryos *brat*^*fs1*^/*Df(2L)TE37C-7* females were collected from a cross of *brat*^*fs1*^/*CyO* flies to *Df(2L)TE37C-7/CyO* flies. These females were mated to the males that resulted from the same cross which consisted mostly of *brat*^*fs1*^/*CyO* or *Df(2L)TE37C-7*/*CyO* since *trans*-heterozygotes were produced at a very low frequency. The resulting embryos failed to hatch and showed reduced numbers of abdominal segments (data not shown) consistent with the *brat* mutant phenotype [3]. Embryos from *w*^*1118*^ flies served as the wild-type control. Embryos were collected at 0 to 1.5, 1.5 to 3.0, 3.0 to 4.5, and 4.5 to 6.0 h post egg-laying, dechorionated in bleach and washed with 0.1% Triton X-100. Embryos were then disrupted in TRI reagent (Molecular Research Centre, Inc., Cincinnati, OH, USA), debris was removed by centrifugation at 14,000 rpm, 4°C for 15 min, and RNA was isolated following the manufacturer’s protocol.

For microarray analysis of *brat* mutant and *w*^*1118*^ embryo time-course samples, double-stranded cDNA was prepared following the protocol described in the NimbleGen Array User’s Guide (Gene Expression Arrays, version 6.0), but using a primer mixture containing 50 ng/μL random hexamer primers and 67 pmol/μL anchored-oligo-dT primers. Double-stranded cDNA was prepared from 2.5 μg of total RNA for each sample. A total of 500 ng of double-stranded cDNA was labelled with Cy3- or Cy5-tagged random nonamers (TriLink BioTechnologies, Inc.) following the Roche NimbleGen protocol. Labelled cDNA was then hybridized to custom-designed *Drosophila* 12 × 135 K NimbleGen arrays (GEO platform number: GPL8593): each array was hybridized with 1 μg each of one Cy3- and one Cy5-labelled sample, and washed, according to the NimbleGen protocol. Three biological replicates were performed for each time point and each genotype. Arrays were scanned with a GenePix4000B microarray scanner system (Molecular Devices, Inc., Sunnyvale, CA, USA). Scanned images were initially quantified using Nimblescan (Roche) and the resulting data were normalized using the ArrayStar 12 (DNASTAR) software using the RMA quantile method. All samples were normalized together.

To identify mRNAs up- or downregulated in *brat* mutant versus *w*^*1118*^ embryos, microarray data was analyzed using the Significance Analysis of Microarrays (SAM) [73] function available in the MultiExperiment Viewer software application [61,62]. Prior to performing SAM analysis to identify up- and downregulated transcripts, we first defined the set of mRNAs expressed in our embryo samples as follows: for each time point and genotype, we defined the set of expressed transcripts by performing one-class SAM analysis to identify transcripts whose normalized signal intensity was significantly greater than the mean normalized signal intensity of all transcripts represented on the array (FDR <5%). We then took the union of the expressed transcripts for all time points in both genotypes, and defined this as our set of expressed transcripts for the experiment. After filtering the transcripts present on the array to only include this set of expressed transcripts, the normalized microarray signal intensities in the *brat* mutant versus *w*^*1118*^ embryos at each time point were compared, using SAM two-class unpaired analysis. Genes significantly higher or lower in the *brat* mutant compared to *w*^*1118*^, with an FDR <5%, were defined as up- or downregulated in *brat* mutants. The co-expressed unchanged transcripts used in the motif enrichment analysis were those that were defined as expressed but which were less than 1.1-fold higher or lower in *brat* mutants, and with an FDR >50%.

For the purposes of all subsequent analyses, gene IDs were updated to the most recent version by taking the FlyBase FBgn IDs present in the array gene description file and updating them using the ‘Upload/Convert IDs’ tool available on FlyBase, to IDs consistent with FlyBase release 6.03.

### K-means clustering of transcripts upregulated in *brat* mutants

K-means clustering was performed to partition the union of the transcripts defined as upregulated at any time point in *brat* mutant embryos, based only on transcript expression in the four time points in *w*^*1118*^ embryos. This analysis was carried out using algorithms available in the MultiExperiment Viewer software application [61,62]: six clusters were chosen to optimally describe the data based on visual inspection of the Figure of Merit for k-means clustering, and the k-means clustering algorithm was used to perform the clustering, using Pearson correlation as the distance metric. Upon 100 repeated iterations of the k-means clustering algorithm with the number of clusters set to six, carried out using the k-means clustering support tool in the MultiExperiment Viewer software, the same six clusters were generated at least 50% of the time; these were designated as Classes A through F.

### Comparisons of transcripts upregulated in *brat* mutants to other datasets

For comparisons of transcripts upregulated in *brat* mutants to previously published datasets, datasets were obtained either from the publications themselves [52,53,59], or were provided to us by the authors [58,65]. Lists from Thomsen et al. [59] were: the entire set of maternally expressed mRNAs in early embryos; the entire set of maternally expressed unstable mRNAs in early embryos (Classes II through V as defined therein); mRNAs degraded exclusively by the maternal, or early, pathway (Class II ‘exclusively maternally degraded’); mRNAs degraded exclusively by the zygotic, or late, pathway (Class IV ‘exclusively zygotically degraded’); mRNAs degraded by both the maternal, or early, and zygotic, or late, pathways, (Class V ‘both maternally and zygotically degraded’); and the entire set of zygotically synthesized transcripts in early embryos (the union of ‘purely zygotic’ and ‘stable + transcription’). Prior to carrying out the comparisons, all gene IDs were updated using the ‘Upload/Convert IDs’ tool available on FlyBase, to IDs consistent with FlyBase release 6.03. Enrichments and depletions were determined using a two-sided Fisher’s exact test, followed by multiple testing correction using the Benjamini-Hochberg procedure to determine FDRs (using R software). Multiple test corrections were carried out separately for comparisons of each class of upregulated mRNAs to the published datasets. Enrichments or depletions with an FDR less than 5% were considered significant. For comparisons to our BRAT RIP-Chip datasets, tests were carried out with the background set as the intersection of the list of expressed genes defined by our RIP-Chip experiment and the list of expressed genes defined by the time-course microarray experiment. For comparisons to datasets describing maternally- versus zygotically-expressed transcripts, tests were carried out with the background set considered as the list of expressed genes defined for our microarray experiment, as described above, or the intersection of this list of expressed genes and the relevant background set from the publication, where applicable. For comparisons to datasets describing maternally expressed and degraded transcripts, tests were carried out with the background set as the intersection of the list of expressed genes defined by our time-course microarray experiment and the list of all maternally-expressed genes defined by the relevant publication. For comparisons to transcripts dependent on Smaug for their decay, the tests were carried out with the background set as the intersection of the list of expressed genes defined by our time-course microarray experiment and the list of all maternally-expressed and degraded transcripts defined by Tadros *et al.* [60]. For comparisons to transcripts dependent on the *miR-309* cluster of miRNAs for their decay, tests were carried out with the background set as the intersection of the list of expressed genes defined by our time-course microarray experiment and the union of the lists of all maternally-expressed and degraded transcripts from Tadros *et al.* [60], De Renzis *et al.* [58], and Thomsen *et al.* [59]. For comparisons to Zelda transcriptional targets, the tests were carried out with the background set as the intersection of the list of expressed genes defined by our time-course microarray experiment and the union of the lists of all zygotically-expressed transcripts defined by De Renzis *et al.* [58] and Thomsen *et al.* [59].

### Assessment of *hb* RNA stability in *brat* mutant embryos

To assess the stability of the maternally-expressed *hb* transcript (*hb*-RB) in wild-type and *brat* mutant embryos, total RNA was isolated from embryos collected from *w*^*1118*^ or *brat*^*fs1*^/*Df(2L)TE37C-7* females, as described above for the *brat* mutant microarray experiment. A total of 50 ng of total RNA was then used to prepare single-stranded cDNA by reverse transcription using random hexamer primers and Superscript II reverse transcriptase (Invitrogen). The single-stranded cDNA was used to perform quantitative real-time PCR with primers specific to the *hb*-RB transcript, as well as *RpL32* as a control mRNA that is unaffected in *brat* mutants, using SYBR green PCR master mix (ABI) and a CFX384 Real-Time System (Bio-Rad). Relative levels of the *hb*-RB and *RpL32* transcripts at each time point for each genotype were determined using a standard curve.

## Additional files

Additional file 1:
**A figure showing western blots demonstrating that the anti-PUM and anti-BRAT synthetic antibodies successfully IP PUM and BRAT.**
Additional file 2:
**A table listing all BRAT targets passing our FDR (<5%) and fold-enrichment (>1.5-fold enriched) cutoffs, with associated FDRs and enrichments indicated.**
Additional file 3:
**A table listing all PUM targets passing our FDR (<5%) and fold-enrichment (>1.5-fold enriched) cutoffs, with associated FDRs and enrichments indicated.**
Additional file 4:
**A figure showing Venn diagrams comparing PUM-associated mRNAs and BRAT-associated mRNAs with a previously published list of mRNAs associated with transgenically expressed PUM-RBD in whole ovaries [** 37 **].**
Additional file 5:
**A table summarizing the results of the cross-validation analysis for motif discovery based on PUM-associated mRNAs and BRAT-associated mRNAs, including motifs and AUROCs.**
Additional file 6:
**A file summarizing the results of the BRAT RNAcompete experiment, including a list of the nucleotide probabilities associated with the motif calculated from the top 10 7-mers (Figure** 2 **D), as well as a list of the top 100 7-mers.**
Additional file 7:
**A figure showing verification of the BRAT knockdown for S2 tissue culture cell experiments, by RT-qPCR.**
Additional file 8:
**A file containing tables summarizing the results of the gene annotation enrichment analyses, performed using the DAVID functional annotation tool, for all sets and subsets of BRAT and PUM targets.**
Additional file 9:
**A file containing tables summarizing the results of the Fly-FISH annotation enrichment analyses, for all sets and subsets of BRAT and PUM targets.**
Additional file 10:
**A file containing tables summarizing the results of the comparisons of BRAT- and PUM-associated mRNAs with various translation and stability datasets included in Table** 3 **.**
Additional file 11:
**A file containing tables listing the genes whose transcripts are significantly upregulated in** ***brat*** **mutant embryos at each of the four time-points assayed, with FDRs and fold-change values indicated.**
Additional file 12:
**A file containing a table summarizing the results of the comparison of transcripts up- or down-regulated in** ***brat*** **mutant embryos to BRAT-associated mRNAs, and the results of motif enrichment testing for BRAT-binding sites in the 3’UTRs of transcripts up- or downregulated in** ***brat*** **mutant embryos.**
Additional file 13:
**A file containing tables listing the mRNAs in each of the six classes of upregulated transcripts, A through F, generated by k-means clustering.**
Additional file 14:
**Figures showing heat-maps depicting the expression patterns, in wild-type and** ***brat*** **mutant embryos, of the mRNAs in each of the six classes of transcripts upregulated in** ***brat*** **mutants, Classes A through F.**
Additional file 15:
**A file containing tables summarizing the results of the comparisons of the six classes of transcripts upregulated in** ***brat*** **mutant embryos (Classes A through F) to various previously published datasets.**
Additional file 16:
**A file containing tables summarizing the results of the gene annotation enrichment analyses, performed using the DAVID functional annotation tool, for those transcripts upregulated in** ***brat*** **mutant embryos in Classes A through C.**
Additional file 17:
**A file containing tables summarizing the results of the Fly-FISH annotation enrichment analyses, for those transcripts upregulated in** ***brat*** **mutant embryos in Classes A through C.**
Additional file 18:
**A figure showing zygotically expressed** ***hb*** **mRNA (** ***hb-RA*** **) in wild-type and** ***brat*** **-mutant embryos.**

## Abbreviations

AUROC: area under the receiver-operating characteristic
Fabs: antigen-binding fragments
FDR: False discovery rate
GO: Gene Ontology
GPI: glycophosphatidylinositol
miRNAs: microRNAs
MZT: maternal-to-zygotic transition
NRE: NOS response element
ORF: open reading frame
RBD: RNA-binding domain
RBPs: RNA-binding proteins
RIP: RNA co-immunoprecipitations
SAM: Significance Analysis of Microarrays
UTR: untranslated region
