# Supplementary material for: Brain tumor is a sequence-specific RNA-binding protein that directs maternal mRNA clearance during the Drosophila maternal-to-zygotic transition
Source: Genome Biol. 2015 May 12;16(1):94. doi: 10.1186/s13059-015-0659-4 (PMC4460960; doi:10.1186/s13059-015-0659-4)
Supplement: Additional file 1: — A figure showing western blots demonstrating that the anti-PUM and anti-BRAT synthetic antibodies successfully IP PUM and BRAT. [file 13059_2015_659_MOESM1_ESM.pdf]

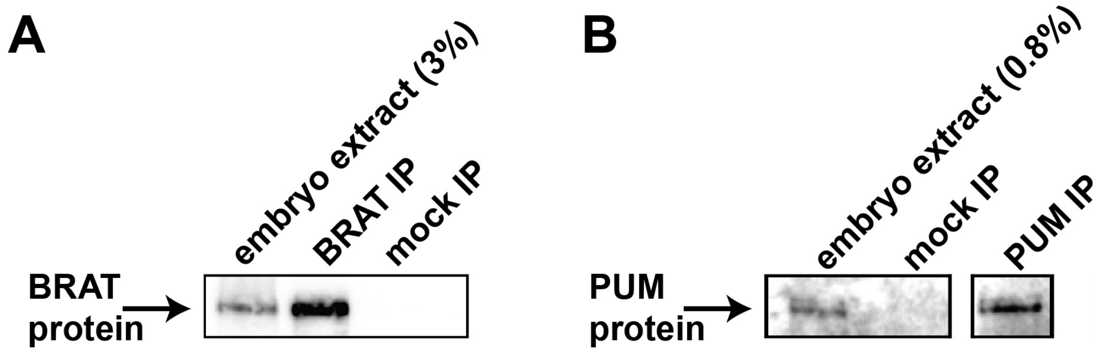


**Additional File 1. Synthetic antibodies against BRAT and PUM immunoprecipitate their target proteins from embryo extract.** Extract prepared from embryos collected 0-to-3 hours post-egg-laying was immunoprecipitated with a synthetic antibody directed against either BRAT **(A)** or PUM **(B)**. In both cases mock immunoprecipitations were performed using the C1 synthetic antibody as a negative control. Immunoprecipitates were analyzed via western blotting with conventional anti-BRAT antibody, provided by Robin Wharton **(A)** or with conventional anti-PUM antibody, provided by Paul Macdonald **(B)**. Embryo extract lanes represent 3% or 0.8% of the material used in the immunoprecipitations in **(A)** and **(B)**, respectively.
