# Supplementary material for: Brain tumor is a sequence-specific RNA-binding protein that directs maternal mRNA clearance during the Drosophila maternal-to-zygotic transition
Source: Genome Biol. 2015 May 12;16(1):94. doi: 10.1186/s13059-015-0659-4 (PMC4460960; doi:10.1186/s13059-015-0659-4)
Supplement: Additional file 4: — A figure showing Venn diagrams comparing PUM-associated mRNAs and BRAT-associated mRNAs with a previously published list of mRNAs associated with transgenically expressed PUM-RBD in whole ovaries [ 37 ]. [file 13059_2015_659_MOESM4_ESM.pdf]

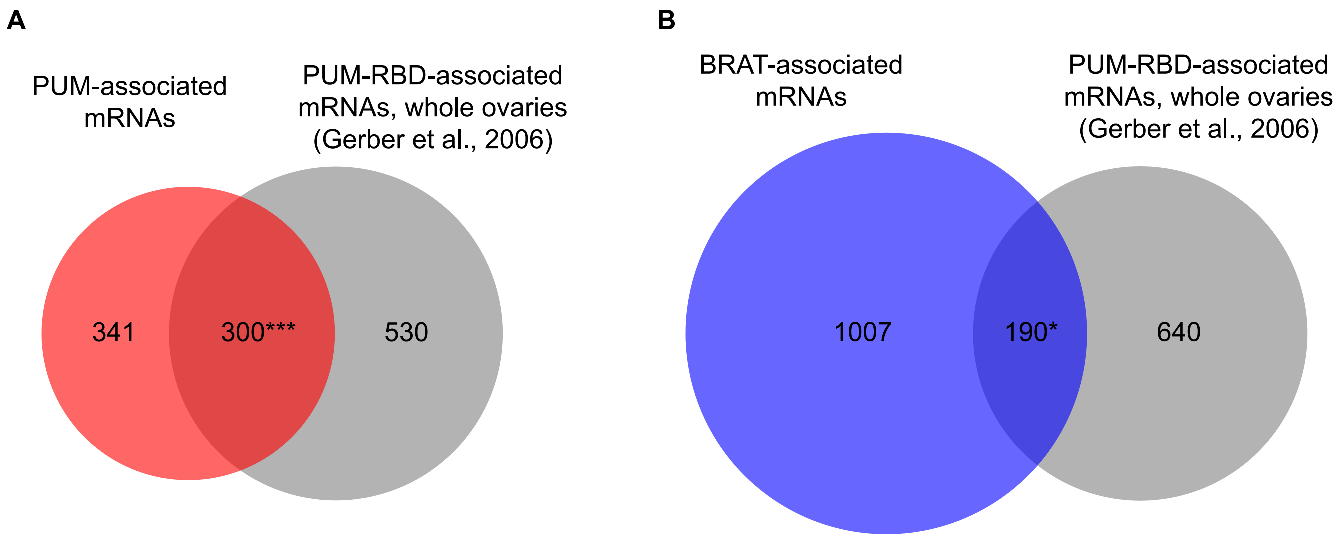

Additional File 4. Venn diagrams comparing previously reported PUM-RBD-associated mRNAs from whole ovaries (Gerber et al., 2006) to (A) PUM-associated mRNAs and (B) BRAT-associated mRNAs in early embryos, as determined by our RIP-Chip experiments. ***Fisher's exact test *P*-value = 8.22×10^{-110}; *Fisher's exact test *P*-value = 6.11×10^{-3}.
