# Supplementary material for: Brain tumor is a sequence-specific RNA-binding protein that directs maternal mRNA clearance during the Drosophila maternal-to-zygotic transition
Source: Genome Biol. 2015 May 12;16(1):94. doi: 10.1186/s13059-015-0659-4 (PMC4460960; doi:10.1186/s13059-015-0659-4)
Supplement: Additional file 7: — A figure showing verification of the BRAT knockdown for S2 tissue culture cell experiments, by RT-qPCR. [file 13059_2015_659_MOESM7_ESM.pdf]

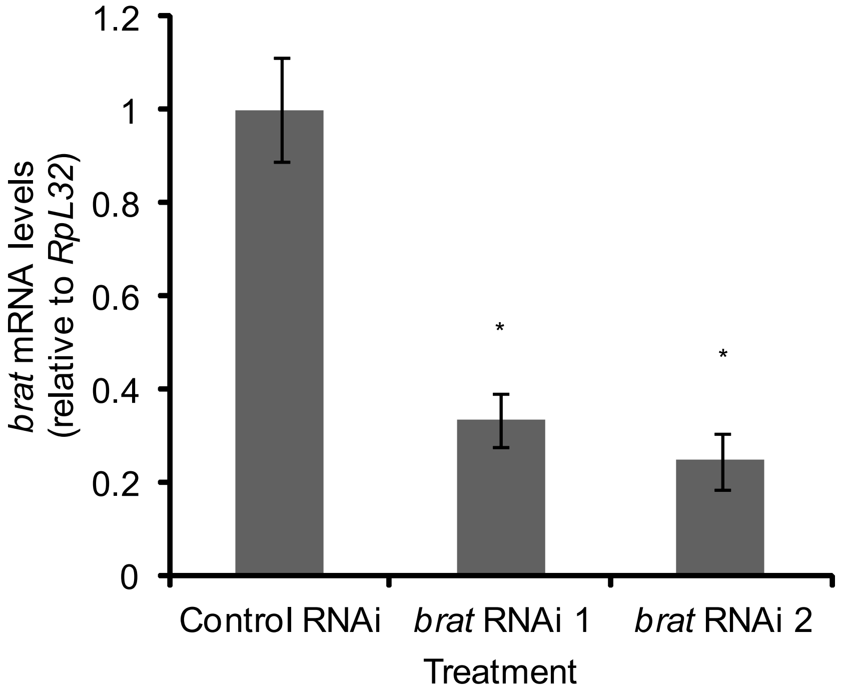

Additional File 7. Quantitation of *brat* mRNA levels, normalized to *RpL32* mRNA levels, after treatment with either control dsRNA, or one of two dsRNAs against *brat*. Knockdown reduced *brat* mRNA expression to approximately 25-35% of levels in control dsRNA treatment (values represent average of two biological replicates +/- standard deviation). *P < 0.05 (Student's t-test).
