# Supplementary material for: Brain tumor is a sequence-specific RNA-binding protein that directs maternal mRNA clearance during the Drosophila maternal-to-zygotic transition
Source: Genome Biol. 2015 May 12;16(1):94. doi: 10.1186/s13059-015-0659-4 (PMC4460960; doi:10.1186/s13059-015-0659-4)
Supplement: Additional file 14: — Figures showing heat-maps depicting the expression patterns, in wild-type and brat mutant embryos, of the mRNAs in each of the six classes of transcripts upregulated in brat mutants, Classes A through F. [file 13059_2015_659_MOESM14_ESM.pdf]

Class A

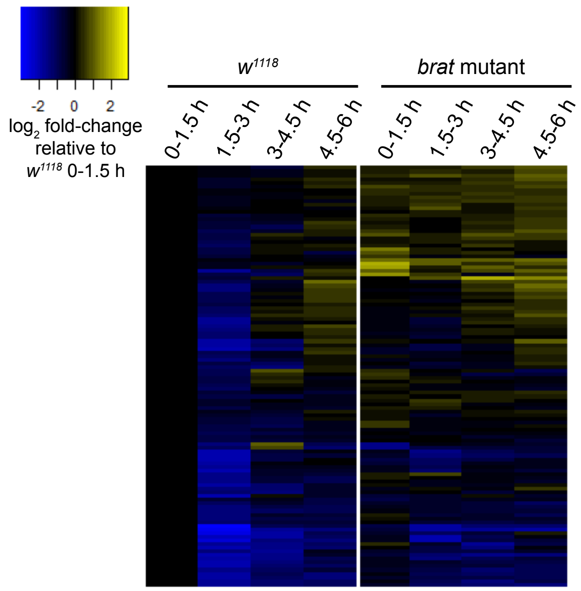

Class B

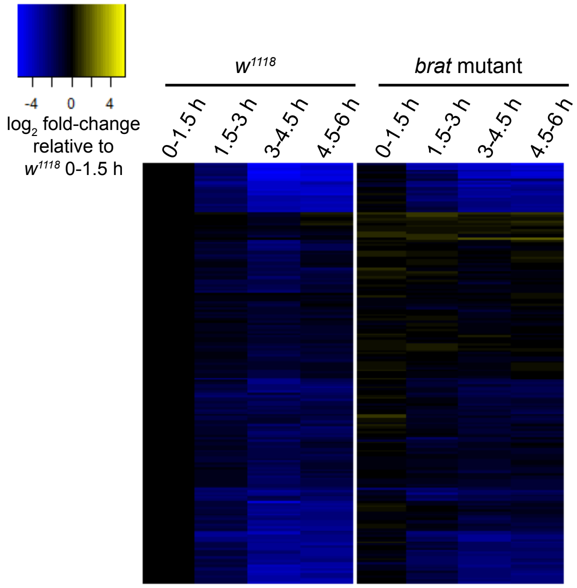

Class C

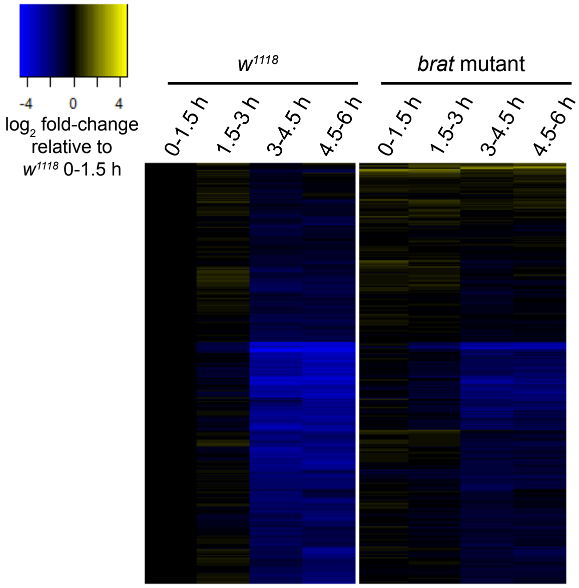

Class D

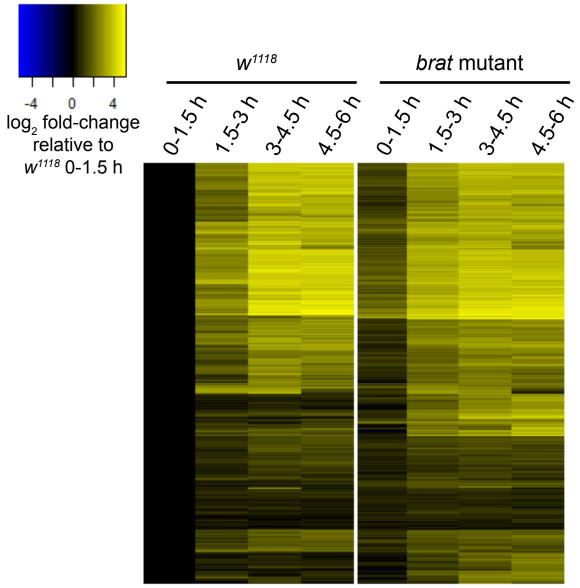

Class E

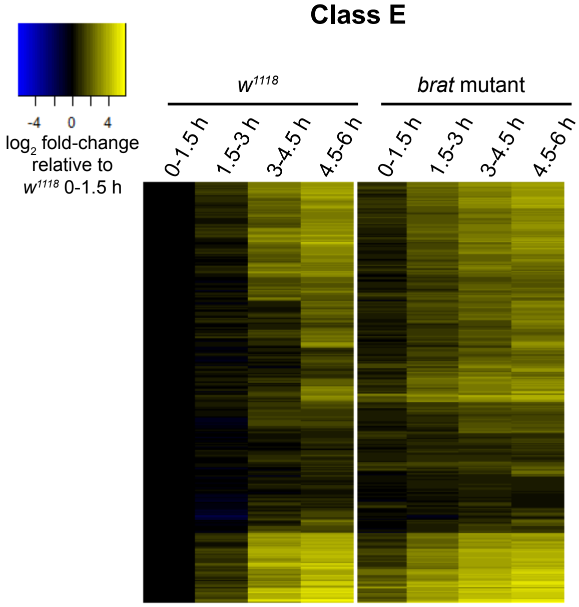

Class F

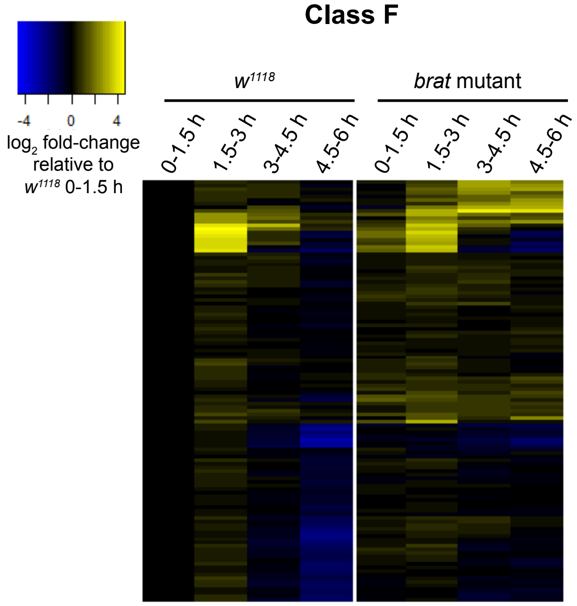

Additional File 14. Heatmaps depicting the expression over 0-to-6 hours, in wild-type and *brat* mutant embryos, of mRNAs in each of the six classes (A through F) of transcripts upregulated in *brat* mutants. Individual rows represent different transcripts, and expression values for each transcript at all time-points in both genotypes were normalized to levels in 0-to-1.5 h wild-type embryos. The scale therefore represents log_2 fold-changes in expression relative to 0-to-1.5 h wild-type embryos.
