# Supplementary material for: Brain tumor is a sequence-specific RNA-binding protein that directs maternal mRNA clearance during the Drosophila maternal-to-zygotic transition
Source: Genome Biol. 2015 May 12;16(1):94. doi: 10.1186/s13059-015-0659-4 (PMC4460960; doi:10.1186/s13059-015-0659-4)
Supplement: Additional file 18: — A figure showing zygotically expressed hb mRNA ( hb-RA ) in wild-type and brat -mutant embryos. [file 13059_2015_659_MOESM18_ESM.pdf]

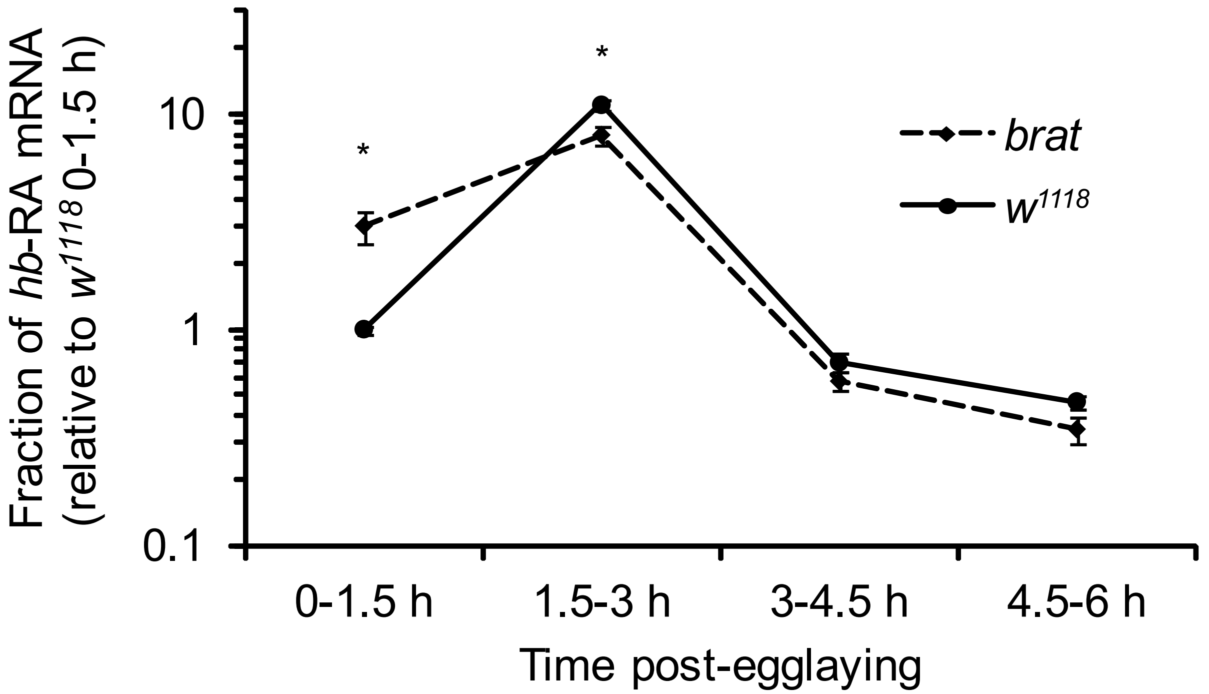

Additional File 18. Expression of zygotically expressed *hb* mRNA (*hb*-RA) in wild-type (w^{1118}) and *brat*-mutant embryos. Levels of *hb*-RA were assayed by RT-qPCR in *brat* mutant or wild-type embryos collected 0-to-1.5, 1.5-to-3.0, 3.0-to-4.5, and 4.5-to-6.0 hours post-egglaying, and normalized to levels of *RpL32* mRNA, whose levels are stable throughout this time-course. *hb*-RA mRNA levels were significantly higher in *brat* mutant than wild-type embryos at 0-to-1.5 but lower at 1.5-to-3.0 hours (values represent average of three biological replicates +/- standard error of the mean). Note that expression of *hb*-RA is transcriptionally activated by Zelda via enhancement of Bicoid binding to the *hb* gene [54]. Thus, the changes in expression in the *brat* mutant versus the wild type may be a consequence of changes in stability of the *hb*-RA mRNA directed by BRAT itself and/or upregulation of transcription of the *hb*-RA mRNA, the latter because of higher levels of Zelda. This caveat does not apply to our analyses of maternal *hb* mRNA shown in Figure 9. **P* < 0.05 (Student's t-test).
